# Supplementary material for: Data-driven learning how oncogenic gene expression locally alters heterocellular networks
Source: Nat Commun. 2022 Apr 13;13:1986. doi: 10.1038/s41467-022-29636-3 (PMC9007999; doi:10.1038/s41467-022-29636-3)
Supplement: Supplementary file 1 — Supplementary Information [file 41467_2022_29636_MOESM1_ESM.pdf]

Data-driven learning how oncogenic gene expression locally alters heterocellular networks

David J. Klinke II[1,2,3], Audry Fernandez[2,3], Wentao Deng[2,3], Atefeh Razazan[2,3], Habibolla Latifizadeh[4], and Anika C. Pirkey[1]
[1] Department of Chemical and Biomedical Engineering, West Virginia University, Morgantown, WV
[2] Department of Microbiology, Immunology and Cell Biology, West Virginia University, Morgantown, WV
[3] WVU Cancer Institute, West Virginia University, Morgantown, WV
[4] School of Mathematical and Data Sciences, West Virginia University, Morgantown, WV

Table of contents.

Page	Label	Description
2	Supplementary Table 1	List of edges and corresponding arcs, whether an arc was predicted to promote (+) or inhibit (-) the target node, and the strength inferred using the different structure learning algorithms in analyzing the features present in TCGA breast cancer dataset.
3	Supplementary Table 2	List of edges and corresponding arcs, whether an arc was predicted to promote (+) or inhibit (-) the target node, and the strength inferred using the different structure learning algorithms in analyzing the features present in dataset containing common melanocytic nevi and primary melanoma tissue samples (GEO).
4	Supplementary Table 3	List of edges and corresponding arcs, whether an arc was predicted to promote (+) or inhibit (-) the target node, and the strength inferred using the different structure learning algorithms in analyzing the features present in primary melanoma tissue samples in the TCGA SKCM dataset.
5	Supplementary Table 4	Proliferation metrics associated $CD4^+$ and $CD8^+$ T cells stimulated in vitro in different conditions.
5	Supplementary Table 5	List of fluorophore-conjugated anti-mouse antibodies using to quantify cell subsets by flow cytometry.
5	Supplementary Table 6	List of primers used for RT-PCR.
6	Supplementary Figure 1	Analysis workflow for Bayesian Network Inference.
7	Supplementary Figure 2	Impact of arcs included in blacklist on consensus edges in the seed network.
8	Supplementary Figure 3	Summary of the evidence obtained from two melanoma datasets supporting the consensus edges in the seed network.
9	Supplementary Figure 4	Conditional probability query of the SKCM DAG compared against digital cytometry estimates obtained from experimental data.
10	Supplementary Figure 5	Flow cytometry gating strategy for T cells.
10	Supplementary Figure 6	Flow cytometry gating strategy for B, NK, and NKT cells.
11	Supplementary Figure 7	Flow cytometry gating strategy for Tumor associated neutrophils and myeloid cell subsets.
12	Supplementary Figure 8	Distribution in digital cytometry features extracted from the BRCA dataset stratified into quantiles based on CCN4 expression.
12	Supplementary Figure 9	Distribution in digital cytometry features extracted from the SKCM dataset stratified into quantiles based on CCN4 expression.
13	Supplementary Figure 10	Control experiments related to ELISpot assay using an inducible CCN4 YUMM1.7 cell line.

Supplementary Table 1. List of edges and corresponding arcs, whether an arc was predicted to promote (+) or inhibit (-) the target node, and the strength inferred using the different structure learning algorithms in analyzing the features present in TCGA breast cancer dataset. Rows highlighted in blue indicate arcs that were included in the consensus seed network (a.k.a. “whitelist"), yellow indicate that the directionality was unclear, and red indicate arcs included in the “blacklist". The edge numbers correspond to the x-axis in Figures 2 and Supplementary Figure 2.

Edge No	from	to	mmpc CorSign	hiton CorSign	pc_stable strength	pcstable CorSign	gs strength	gs CorSign	iamb strength	iamb CorSign	iambfdr strength	iambfdr CorSign	tabu strength	tabu CorSign	hc strength	hc CorSign	mmhc strength	mmhc CorSign	rsmax2 strength	rsmax2 CorSign	Hit Count	Min strength	Max strength
1	CCN4	Mesenchymal	+	+	1.79E-139	+	2.02E-138	+	2.02E-138	+	2.02E-138	+	1.95E-148	+	1.95E-148	+	1.79E-139	+	1.79E-139	+	10	1.95E-148	2.02E-138
1	Mesenchymal	CCN4	+	+																			
2	pM0	pM1	-	-									3.77E-48	-	3.77E-48	-					10	0.00E+00	3.77E-48
2	pM1	pM0	-	-	0.00E+00	-	0.00E+00	-	0.00E+00	-	0.00E+00	-					0.00E+00	-	0.00E+00	-			
3	pM0	pM2	-	-									0.00E+00	-	0.00E+00	-					10	0.00E+00	0.00E+00
3	pM2	pM0	-	-	0.00E+00	-	0.00E+00	-	0.00E+00	-	0.00E+00	-					0.00E+00	-	0.00E+00	-			
4	CAF_lg	Mesenchymal	+	+																	10	1.86E-108	9.62E-73
4	Mesenchymal	CAF_lg	+	+	8.08E-86	+	9.62E-73	+	9.62E-73	+	9.62E-73	+	2.94E-97	+	1.86E-108	+	9.56E-73	+	2.35E-86	+			
5	Cancer	CCN4	+	+	2.06E-55	+	2.06E-55	+	2.06E-55	+	2.06E-55	+	2.06E-55	+	2.06E-55	+	2.06E-55	+	2.06E-55	+	10	2.06E-55	2.06E-55
5	CCN4	Cancer	+	+																			
6	NK.cells.active_lg	NK.cells.rest_lg	-	-	4.80E-30	-	4.80E-30	-	4.80E-30	-	4.80E-30	-	1.15E-33	-	1.15E-33	-	4.80E-30	-	4.80E-30	-	10	1.15E-33	4.80E-30
6	NK.cells.rest_lg	NK.cells.active_lg	-	-																			
7	B.cells.naive_lg	T.cells.CD8_lg	+	+	2.92E-27	+	9.25E-27	+	9.25E-27	+	2.92E-27	+	3.10E-16	+	3.10E-16	+	2.92E-27	+	2.92E-27	+	10	2.92E-27	3.10E-16
7	T.cells.CD8_lg	B.cells.naive_lg	+	+																			
8	CAF_lg	proliferation	-	-											9.87E-15	-	7.61E-26	-	7.61E-26	-	10	7.61E-26	3.37E-12
8	proliferation	CAF_lg	-	-	1.80E-18	-	6.37E-21	-	6.37E-21	-	6.37E-21	-	3.37E-12	-									
9	Endothelial.cells_lg	proliferation	-	-									5.75E-10	-	7.36E-09	-					10	9.05E-26	7.36E-09
9	proliferation	Endothelial.cells_lg	-	-	6.23E-18	-	9.05E-26	-	2.88E-15	-	9.05E-26	-					2.88E-15	-	2.88E-15	-			
10	Cancer	Epithelial	-	-	1.66E-25	-	1.66E-25	-	1.66E-25	-	1.66E-25	-	1.66E-25	-	1.66E-25	-	1.66E-25	-	1.66E-25	-	10	1.66E-25	1.66E-25
10	Epithelial	Cancer	-	-																			
11	Cancer	proliferation	+	+	1.93E-08	+	6.68E-23	+	6.68E-23	+	6.68E-23	+	4.70E-12	+	1.09E-07	+	3.72E-10	+	3.72E-10	+	10	6.68E-23	1.09E-07
11	proliferation	Cancer	+	+																			
12	CD4Tcell_sc_lg	Macrophages_sc_lg	+	+																	10	1.91E-16	2.01E-13
12	Macrophages_sc_lg	CD4Tcell_sc_lg	+	+	2.01E-13	+	1.91E-16	+	1.91E-16	+	5.09E-16	+	3.07E-14	+	3.07E-14	+	5.09E-16	+	5.09E-16	+			
13	pM1	T.cells.CD8_lg	+	+	3.53E-14	+	8.18E-13	+	8.18E-13	+	3.53E-14	+	8.98E-15	+	8.98E-15	+	3.53E-14	+	3.53E-14	+	10	8.98E-15	8.18E-13
13	T.cells.CD8_lg	pM1	+	+																			
14	Macrophages_sc_lg	T.cells.CD8_lg	+	+	2.38E-12	+	9.15E-13	+	9.15E-13	+	2.38E-12	+	6.56E-12	+	6.56E-12	+	2.38E-12	+	2.38E-12	+	10	9.15E-13	6.56E-12
14	T.cells.CD8_lg	Macrophages_sc_lg	+	+																			
15	CD4Tcell_sc_lg	Endothelial.cells_lg	+	+																	10	3.64E-10	7.84E-07
15	Endothelial.cells_lg	CD4Tcell_sc_lg	+	+	7.84E-07	+	5.65E-08	+	5.65E-08	+	2.93E-07	+	3.64E-10	+	3.64E-10	+	2.93E-07	+	2.93E-07	+			
16	CAF_lg	T.cells.CD8_lg	-	-	3.06E-07	-	2.84E-07	-	2.84E-07	-	3.06E-07	-	3.42E-09	-	3.42E-09	-	3.06E-07	-	3.06E-07	-	10	3.42E-09	3.06E-07
16	T.cells.CD8_lg	CAF_lg	-	-																			
17	B.cells.naive_lg	CD4Tcell_sc_lg	+	+	7.31E-05	+	2.95E-06	+	2.95E-06	+	1.95E-06	+	2.17E-07	+	2.17E-07	+	1.95E-06	+	1.95E-06	+	10	2.17E-07	7.31E-05
17	CD4Tcell_sc_lg	B.cells.naive_lg	+	+																			
18	Epithelial	Neutrophils_lg	-	-	6.79E-04	-	2.82E-03	-	2.82E-03	-			1.62E-03	-	1.62E-03	-	6.79E-04	-	6.79E-04	-	9	6.79E-04	2.82E-03
18	Neutrophils_lg	Epithelial	-	-																			
19	Cancer	Endothelial.cells_lg	-	-	2.10E-05	-			7.40E-09	-			3.67E-38	-	3.67E-38	-	7.40E-09	-	7.40E-09	-	8	3.67E-38	2.10E-05
19	Endothelial.cells_lg	Cancer	-	-																			
20	B.cells.naive_lg	Endothelial.cells_lg	+	+	4.01E-20	+															8	2.71E-22	1.52E-18
20	Endothelial.cells_lg	B.cells.naive_lg	+	+			2.71E-22	+	2.71E-22	+	2.71E-22	+					1.52E-18	+	2.71E-22	+			
21	Cancer	pM2	-	-	1.91E-42	-							7.00E-04	-	7.00E-04	-	1.91E-42	-	1.91E-42	-	7	1.91E-42	7.00E-04
21	pM2	Cancer	-	-																			
22	Cancer	Mesenchymal	+	+	1.16E-16	+							8.89E-17	+	8.89E-17	+	1.16E-16	+	1.16E-16	+	7	8.89E-17	1.16E-16
22	Mesenchymal	Cancer	+	+																			
23	pM2	proliferation	-	-	3.30E-10	-							1.02E-10	-	6.65E-10	-	2.09E-11	-	2.09E-11	-	7	2.09E-11	6.65E-10
23	proliferation	pM2	-	-																			
24	Neutrophils_lg	pM2	+	+																	7	7.51E-09	1.57E-04
24	pM2	Neutrophils_lg	+	+	1.57E-04	+							7.51E-09	+	7.51E-09	+	1.57E-04	+	1.57E-04	+			
25	Macrophages_sc_lg	Neutrophils_lg	+	+	1.86E-03	+	1.59E-03	+	1.59E-03	+							1.86E-03	+	1.86E-03	+	7	1.59E-03	1.86E-03
25	Neutrophils_lg	Macrophages_sc_lg	+	+																			
26	CAF_lg	pM1	-	-																	6	3.74E-24	4.37E-12
26	pM1	CAF_lg	-	-	1.06E-21	-							4.37E-12	-	2.49E-13	-			3.74E-24	-			
27	pM1	pM2											0.00E+00	-	0.00E+00	-					5	0.00E+00	3.43E-04
27	pM2	pM1					3.43E-04	-	3.43E-04	-	3.43E-04	-											
28	NK.cells.rest_lg	T.cells.CD8_lg					1.24E-02	-	1.24E-02	-			7.88E-04	-	7.88E-04	-					4	7.88E-04	1.24E-02
28	T.cells.CD8_lg	NK.cells.rest_lg																					
29	CD4Tcell_sc_lg	NK.cells.active_lg																			4	1.08E-03	1.26E-03
29	NK.cells.active_lg	CD4Tcell_sc_lg					1.26E-03	-	1.26E-03	-			1.08E-03	-	1.08E-03	-							
30	CD4Tcell_sc_lg	pM1	+	+																	3	1.23E-02	1.23E-02
30	pM1	CD4Tcell_sc_lg	+	+	1.23E-02	+																	
31	Cancer	pM1											2.35E-35	+	2.35E-35	+					2	2.35E-35	2.35E-35
31	pM1	Cancer																					
32	Cancer	pM0											5.03E-25	+	5.03E-25	+					2	5.03E-25	5.03E-25
32	pM0	Cancer																					
33	Endothelial.cells_lg	Mesenchymal																			2	4.02E-19	4.02E-19
33	Mesenchymal	Endothelial.cells_lg											4.02E-19	+	4.02E-19	+							
34	B.cells.naive_lg	pM1											4.18E-19	+	4.18E-19	+					2	4.18E-19	4.18E-19
34	pM1	B.cells.naive_lg																					
35	CAF_lg	Macrophages_sc_lg																			2	5.33E-17	1.86E-15
35	Macrophages_sc_lg	CAF_lg											5.33E-17	-	1.86E-15	-							
36	B.cells.naive_lg	Cancer																			2	3.36E-13	4.79E-07
36	Cancer	B.cells.naive_lg											3.36E-13	-	4.79E-07	-							
37	CAF_lg	pM2																			2	8.24E-13	3.71E-04
37	pM2	CAF_lg											3.71E-04	+	8.24E-13	+							
38	Mesenchymal	pM0																			2	1.51E-12	1.51E-12
38	pM0	Mesenchymal											1.51E-12	+	1.51E-12	+							
39	Endothelial.cells_lg	pM1																			2	4.23E-12	4.23E-12
39	pM1	Endothelial.cells_lg											4.23E-12	+	4.23E-12	+							
40	CCN4	proliferation											7.29E-12	-	1.51E-01	-					2	7.29E-12	1.51E-01
40	proliferation	CCN4																					
41	Cancer	T.cells.CD8_lg											2.30E-10	-	2.30E-10	-					2	2.30E-10	2.30E-10
41	T.cells.CD8_lg	Cancer																					
42	Macrophages_sc_lg	pM0											2.14E-08	-	2.14E-08	-					2	2.14E-08	2.14E-08
42	pM0	Macrophages_sc_lg																					
43	NK.cells.rest_lg	pM0											2.12E-07	+	2.12E-07	+					2	2.12E-07	2.12E-07
43	pM0	NK.cells.rest_lg																					
44	Mesenchymal	Neutrophils_lg											2.39E-07	+	2.39E-07	+					2	2.39E-07	2.39E-07
44	Neutrophils_lg	Mesenchymal																					
45	CCN4	Macrophages_sc_lg											4.84E-07	+	4.84E-07	+					2	4.84E-07	4.84E-07
45	Macrophages_sc_lg	CCN4																					
46	Cancer	NK.cells.rest_lg											4.25E-06	-	4.25E-06	-					2	4.25E-06	4.25E-06
46	NK.cells.rest_lg	Cancer																					
47	CD4Tcell_sc_lg	Epithelial																			2	9.98E-06	9.98E-06
47	Epithelial	CD4Tcell_sc_lg											9.98E-06	-	9.98E-06	-							
48	B.cells.naive_lg	NK.cells.rest_lg											3.72E-05	-	3.72E-05	-					2	3.72E-05	3.72E-05
48	NK.cells.rest_lg	B.cells.naive_lg																					
49	Epithelial	Mesenchymal											7.35E-05	+	7.35E-05	+					2	7.35E-05	7.35E-05
49	Mesenchymal	Epithelial																					
50	Endothelial.cells_lg	Neutrophils_lg											7.61E-05	-	7.61E-05	-					2	7.61E-05	7.61E-05
50	Neutrophils_lg	Endothelial.cells_lg																					
51	Macrophages_sc_lg	proliferation											1.53E-01	-	1.18E-04	-					2	1.18E-04	1.53E-01
51	proliferation	Macrophages_sc_lg																					
52	Mesenchymal	T.cells.CD8_lg											2.04E-04	+	2.04E-04	+					2	2.04E-04	2.04E-04
52	T.cells.CD8_lg	Mesenchymal																					
53	CCN4	NK.cells.active_lg											2.64E-04	-	2.64E-04	-					2	2.64E-04	2.64E-04
53	NK.cells.active_lg	CCN4																					
54	CAF_lg	Epithelial																			2	5.82E-04	2.94E-03
54	Epithelial	CAF_lg											2.94E-03	+	5.82E-04	+							
55	B.cells.naive_lg	CAF_lg											1.48E-03	-	6.27E-03	-					2	1.48E-03	6.27E-03
55	CAf_lg	B.cells.naive_lg																					
56	NK.cells.rest_lg	proliferation											2.80E-02	+	2.15E-03	+					2	2.15E-03	2.80E-02
56	proliferation	NK.cells.rest_lg																					
57	B.cells.naive_lg	Epithelial	+																		2	8.19E-03	8.19E-03
57	Epithelial	B.cells.naive_lg	+														8.19E-03	+					
58	B.cells.naive_lg	CCN4																			1	5.87E-08	5.87E-08
58	CCN4	B.cells.naive_lg											5.87E-08	+									
59	Cancer	Macrophages_sc_lg																			0	1.00E+00	1.00E+00
59	Macrophages_sc_lg	Cancer																					
60	CAF_lg	Endothelial.cells_lg																			0	1.00E+00	1.00E+00
60	Endothelial.cells_lg	CAF_lg																					
61	CCN4	pM0																			0	1.00E+00	1.00E+00
61	pM0	CCN4																					
62	Endothelial.cells_lg	T.cells.CD8_lg																			0	1.00E+00	1.00E+00
62	T.cells.CD8_lg	Endothelial.cells_lg																					
63	Macrophages_sc_lg	Mesenchymal																			0	1.00E+00	1.00E+00
63	Mesenchymal	Macrophages_sc_lg																					
64	B.cells.naive_lg	Macrophages_sc_lg																			0	1.00E+00	1.00E+00
64	Macrophages_sc_lg	B.cells.naive_lg																					
65	Mesenchymal	pM2																			0	1.00E+00	1.00E+00
65	pM2	Mesenchymal																					
66	NK.cells.active_lg	pM0																			0	1.00E+00	1.00E+00
66	pM0	NK.cells.active_lg																					
67	B.cells.naive_lg	Mesenchymal																			0	1.00E+00	1.00E+00
67	Mesenchymal	B.cells.naive_lg																					
68	CCN4	Endothelial.cells_lg																			0	1.00E+00	1.00E+00
68	Endothelial.cells_lg	CCN4																					
69	CAF_lg	Cancer																			0	1.00E+00	1.00E+00
69	Cancer	CAF_lg																					
70	Neutrophils_lg	pM0																			0	1.00E+00	1.00E+00
70	pM0	Neutrophils_lg																					
71	CCN4	Neutrophils_lg																			0	1.00E+00	1.00E+00
71	Neutrophils_lg	CCN4																					
72	Neutrophils_lg	pM1																			0	1.00E+00	1.00E+00
72	pM1	Neutrophils_lg																					
73	Macrophages_sc_lg	NK.cells.rest_lg																			0	1.00E+00	1.00E+00
73	NK.cells.rest_lg	Macrophages_sc_lg																					
74	Epithelial	pM2																			0	1.00E+00	1.00E+00
74	pM2	Epithelial																					
75	Endothelial.cells_lg	pM0																			0	1.00E+00	1.00E+00
75	pM0	Endothelial.cells_lg																					
76	CCN4	NK.cells.rest_lg																			0	1.00E+00	1.00E+00
76	NK.cells.rest_lg	CCN4																					
77	NK.cells.active_lg	T.cells.CD8_lg																			0	1.00E+00	1.00E+00
77	T.cells.CD8_lg	NK.cells.active_lg																					
78	CAF_lg	CD4Tcell_sc_lg																			0	1.00E+00	1.00E+00
78	CD4Tcell_sc_lg	CAF_lg																					
79	CAF_lg	CCN4																			0	1.00E+00	1.00E+00
79	CCN4	CAF_lg																					
80	Endothelial.cells_lg	Epithelial																			0	1.00E+00	1.00E+00
80	Epithelial	Endothelial.cells_lg																					
81	Macrophages_sc_lg	NK.cells.active_lg																			0	1.00E+00	1.00E+00
81	NK.cells.active_lg	Macrophages_sc_lg																					
82	Macrophages_sc_lg	pM1																			0	1.00E+00	1.00E+00
82	pM1	Macrophages_sc_lg																					
83	Cancer	NK.cells.active_lg																			0	1.00E+00	1.00E+00
83	NK.cells.active_lg	Cancer																					
84	Epithelial	pM0																			0	1.00E+00	1.00E+00
84	pM0	Epithelial																					

Supplementary Table 2. List of edges and corresponding arcs, whether an arc was predicted to promote (+) or inhibit (-) the target node, and the strength inferred using the different structure learning algorithms in analyzing the features present in dataset containing common melanocytic nevi and primary melanoma tissue samples (GEO). Rows highlighted in blue indicate arcs that were included in the consensus seed network (a.k.a. “whitelist"), yellow indicate that the directionality was unclear, and red indicate arcs included in the “blacklist". The edge numbers correspond to the x-axis in Supplementary Figure 3, panel A.

Edge No	from	to	mmpc CorSign	hiton CorSign	pc_stable strength	pcstable CorSign	gs strength	gs CorSign	iamb strength	iamb CorSign	iambfdr strength	iambfdr CorSign	tabu strength	tabu CorSign	hc strength	hc CorSign	mmhc strength	mmhc CorSign	rsmax2 strength	rsmax2 CorSign	Hit Count	Min strength	Max strength
1	CCN4	Mesenchymal	+	+	2.29E-02	+	6.48E-05	+	6.48E-05	+			8.38E-07	+	8.38E-07	+	6.48E-05	+	6.48E-05	+	9	8.38E-07	2.29E-02
1	Mesenchymal	CCN4	+	+																			
2	pM0	pM1											2.28E-02	-	2.28E-02	-					2	2.28E-02	2.28E-02
2	pM1	pM0																					
3	pM0	pM2	-	-	3.31E-38	-	3.31E-38	-	3.31E-38	-	3.31E-38	-	3.31E-38	-	3.31E-38	-	3.31E-38	-	3.31E-38	-	10	3.31E-38	3.31E-38
3	pM2	pM0	-	-																			
4	CAF_lg	Mesenchymal	+	+	2.20E-12	+					6.70E-15	+									10	6.70E-15	5.98E-05
4	Mesenchymal	CAF_lg	+	+			2.75E-14	+	2.75E-14	+			7.46E-12	+	5.98E-05	+	2.75E-14	+	2.75E-14	+			
5	Cancer	CCN4											5.06E-11	+	5.06E-11	+					2	5.06E-11	5.06E-11
5	CCN4	Cancer																					
6	NK.cells.active_lg	NK.cells.rest_lg	-	-													1.02E-06	-			10	1.01E-09	1.68E-05
6	NK.cells.rest_lg	NK.cells.active_lg	-	-	1.68E-05	-	1.01E-09	-	1.01E-09	-	1.68E-05	-	1.01E-09	-	1.01E-09	-			1.68E-05	-			
7	B.cells.ive_lg	T.cells.CD8_lg																			0	1.00E+00	1.00E+00
7	T.cells.CD8_lg	B.cells.ive_lg																					
8	CAF_lg	proliferation																			0	1.00E+00	1.00E+00
8	proliferation	CAF_lg																					
9	Endothelial.cells_lg	proliferation											5.19E-05	-	5.19E-05	-					2	5.19E-05	5.19E-05
9	proliferation	Endothelial.cells_lg																					
10	Cancer	Epithelial	-	-	4.24E-15	-	4.24E-15	-	4.24E-15	-	4.24E-15	-	4.24E-15	-	2.79E-16	-	4.24E-15	-	4.24E-15	-	10	2.79E-16	4.24E-15
10	Epithelial	Cancer	-	-																			
11	Cancer	proliferation					1.47E-09	+	1.47E-09	+			6.39E-11	+	6.39E-11	+					4	6.39E-11	1.47E-09
11	proliferation	Cancer																					
12	CD4Tcell_sc_lg	Macrophages_sc_lg	+	+																	9	4.58E-04	4.58E-04
12	Macrophages_sc_lg	CD4Tcell_sc_lg	+	+	4.58E-04	+	4.58E-04	+	4.58E-04	+			4.58E-04	+	4.58E-04	+	4.58E-04	+	4.58E-04	+			
13	pM1	T.cells.CD8_lg	+	+	3.70E-03	+							5.96E-03	+	5.96E-03	+	3.70E-03	+	3.70E-03	+	7	3.70E-03	5.96E-03
13	T.cells.CD8_lg	pM1	+	+																			
14	Macrophages_sc_lg	T.cells.CD8_lg	+	+	1.79E-04	+	9.32E-06	+	9.32E-06	+	1.82E-11	+	7.68E-06	+	7.68E-06	+	1.79E-04	+	1.79E-04	+	10	1.82E-11	1.79E-04
14	T.cells.CD8_lg	Macrophages_sc_lg	+	+																			
15	CD4Tcell_sc_lg	Endothelial.cells_lg	+	+																	10	8.60E-08	4.15E-07
15	Endothelial.cells_lg	CD4Tcell_sc_lg	+	+	4.15E-07	+	4.15E-07	+	4.15E-07	+	8.60E-08	+	4.15E-07	+	4.15E-07	+	4.15E-07	+	4.15E-07	+			
16	CAF_lg	T.cells.CD8_lg											8.87E-03	+	8.87E-03	+					2	8.87E-03	8.87E-03
16	T.cells.CD8_lg	CAF_lg																					
17	B.cells.ive_lg	CD4Tcell_sc_lg																			0	1.00E+00	1.00E+00
17	CD4Tcell_sc_lg	B.cells.ive_lg																					
18	Epithelial	Neutrophils_lg																			0	1.00E+00	1.00E+00
18	Neutrophils_lg	Epithelial																					
19	Cancer	Endothelial.cells_lg																			0	1.00E+00	1.00E+00
19	Endothelial.cells_lg	Cancer																					
20	B.cells.ive_lg	Endothelial.cells_lg																			2	3.41E-03	3.41E-03
20	Endothelial.cells_lg	B.cells.ive_lg											3.41E-03	+	3.41E-03	+							
21	Cancer	pM2																			0	1.00E+00	1.00E+00
21	pM2	Cancer																					
22	Cancer	Mesenchymal	+	+	2.46E-12	+	6.29E-09	+	6.29E-09	+	2.09E-18	+	8.02E-13	+	8.02E-13	+	6.29E-09	+	6.29E-09	+	10	2.09E-18	6.29E-09
22	Mesenchymal	Cancer	+	+																			
23	pM2	proliferation																			0	1.00E+00	1.00E+00
23	proliferation	pM2																					
24	Neutrophils_lg	pM2																			0	1.00E+00	1.00E+00
24	pM2	Neutrophils_lg																					
25	Macrophages_sc_lg	Neutrophils_lg																			0	1.00E+00	1.00E+00
25	Neutrophils_lg	Macrophages_sc_lg																					
26	CAF_lg	pM1																			0	1.00E+00	1.00E+00
26	pM1	CAF_lg																					
27	pM1	pM2	-	-	2.40E-41	-	2.40E-41	-	2.40E-41	-	2.40E-41	-	2.40E-41	-	2.40E-41	-	2.40E-41	-	2.40E-41	-	10	2.40E-41	2.40E-41
27	pM2	pM1	-	-																			
28	NK.cells.rest_lg	T.cells.CD8_lg																			0	1.00E+00	1.00E+00
28	T.cells.CD8_lg	NK.cells.rest_lg																					
29	CD4Tcell_sc_lg	NK.cells.active_lg																			0	1.00E+00	1.00E+00
29	NK.cells.active_lg	CD4Tcell_sc_lg																					
30	CD4Tcell_sc_lg	pM1																			0	1.00E+00	1.00E+00
30	pM1	CD4Tcell_sc_lg																					
31	Cancer	pM1																			0	1.00E+00	1.00E+00
31	pM1	Cancer																					
32	Cancer	pM0																			0	1.00E+00	1.00E+00
32	pM0	Cancer																					
33	Endothelial.cells_lg	Mesenchymal											2.75E-10	+	2.75E-10	+					2	2.75E-10	2.75E-10
33	Mesenchymal	Endothelial.cells_lg																					
34	B.cells.ive_lg	pM1																			0	1.00E+00	1.00E+00
34	pM1	B.cells.ive_lg																					
35	CAF_lg	Macrophages_sc_lg																			0	1.00E+00	1.00E+00
35	Macrophages_sc_lg	CAF_lg																					
36	B.cells.ive_lg	Cancer																			0	1.00E+00	1.00E+00
36	Cancer	B.cells.ive_lg																					
37	CAF_lg	pM2																			0	1.00E+00	1.00E+00
37	pM2	CAF_lg																					
38	Mesenchymal	pM0																			0	1.00E+00	1.00E+00
38	pM0	Mesenchymal																					
39	Endothelial.cells_lg	pM1																			0	1.00E+00	1.00E+00
39	pM1	Endothelial.cells_lg																					
40	CCN4	proliferation																			0	1.00E+00	1.00E+00
40	proliferation	CCN4																					
41	Cancer	T.cells.CD8_lg																			0	1.00E+00	1.00E+00
41	T.cells.CD8_lg	Cancer																					
42	Macrophages_sc_lg	pM0																			0	1.00E+00	1.00E+00
42	pM0	Macrophages_sc_lg																					
43	NK.cells.rest_lg	pM0	+	+	1.17E-03	+							2.22E-04	+	2.22E-04	+					7	6.30E-05	1.17E-03
43	pM0	NK.cells.rest_lg	+	+													6.30E-05	+	1.17E-03	+			
44	Mesenchymal	Neutrophils_lg																			0	1.00E+00	1.00E+00
44	Neutrophils_lg	Mesenchymal																					
45	CCN4	Macrophages_sc_lg																			0	1.00E+00	1.00E+00
45	Macrophages_sc_lg	CCN4																					
46	Cancer	NK.cells.rest_lg																			0	1.00E+00	1.00E+00
46	NK.cells.rest_lg	Cancer																					
47	CD4Tcell_sc_lg	Epithelial																			0	1.00E+00	1.00E+00
47	Epithelial	CD4Tcell_sc_lg																					
48	B.cells.ive_lg	NK.cells.rest_lg																			0	1.00E+00	1.00E+00
48	NK.cells.rest_lg	B.cells.ive_lg																					
49	Epithelial	Mesenchymal																			0	1.00E+00	1.00E+00
49	Mesenchymal	Epithelial																					
50	Endothelial.cells_lg	Neutrophils_lg																			0	1.00E+00	1.00E+00
50	Neutrophils_lg	Endothelial.cells_lg																					
51	Macrophages_sc_lg	proliferation																			0	1.00E+00	1.00E+00
51	proliferation	Macrophages_sc_lg																					
52	Mesenchymal	T.cells.CD8_lg																			0	1.00E+00	1.00E+00
52	T.cells.CD8_lg	Mesenchymal																					
53	CCN4	NK.cells.active_lg																			0	1.00E+00	1.00E+00
53	NK.cells.active_lg	CCN4																					
54	CAF_lg	Epithelial																			0	1.00E+00	1.00E+00
54	Epithelial	CAF_lg																					
55	B.cells.ive_lg	CAF_lg																			0	1.00E+00	1.00E+00
55	CAf_lg	B.cells.ive_lg																					
56	NK.cells.rest_lg	proliferation																			0	1.00E+00	1.00E+00
56	proliferation	NK.cells.rest_lg																					
57	B.cells.ive_lg	Epithelial																			0	1.00E+00	1.00E+00
57	Epithelial	B.cells.ive_lg																					
58	B.cells.ive_lg	CCN4																			0	1.00E+00	1.00E+00
58	CCN4	B.cells.ive_lg																					
59	Cancer	Macrophages_sc_lg																			0	1.00E+00	1.00E+00
59	Macrophages_sc_lg	Cancer																					
60	CAF_lg	Endothelial.cells_lg			3.11E-06	+															3	3.11E-06	8.04E-04
60	Endothelial.cells_lg	CAF_lg											8.04E-04	+	4.19E-04	+							
61	CCN4	pM0																			0	1.00E+00	1.00E+00
61	pM0	CCN4																					
62	Endothelial.cells_lg	T.cells.CD8_lg																			0	1.00E+00	1.00E+00
62	T.cells.CD8_lg	Endothelial.cells_lg																					
63	Macrophages_sc_lg	Mesenchymal																			2	7.24E-09	7.24E-09
63	Mesenchymal	Macrophages_sc_lg											7.24E-09	+	7.24E-09	+							
64	B.cells.ive_lg	Macrophages_sc_lg																			0	1.00E+00	1.00E+00
64	Macrophages_sc_lg	B.cells.ive_lg																					
65	Mesenchymal	pM2																			0	1.00E+00	1.00E+00
65	pM2	Mesenchymal																					
66	NK.cells.active_lg	pM0																			0	1.00E+00	1.00E+00
66	pM0	NK.cells.active_lg																					
67	B.cells.ive_lg	Mesenchymal																			0	1.00E+00	1.00E+00
67	Mesenchymal	B.cells.ive_lg																					
68	CCN4	Endothelial.cells_lg																			0	1.00E+00	1.00E+00
68	Endothelial.cells_lg	CCN4																					
69	CAF_lg	Cancer																			0	1.00E+00	1.00E+00
69	Cancer	CAF_lg																					
70	Neutrophils_lg	pM0																			0	1.00E+00	1.00E+00
70	pM0	Neutrophils_lg																					
71	CCN4	Neutrophils_lg											7.22E-04	+	7.22E-04	+					2	7.22E-04	7.22E-04
71	Neutrophils_lg	CCN4																					
72	Neutrophils_lg	pM1																			0	1.00E+00	1.00E+00
72	pM1	Neutrophils_lg																					
73	Macrophages_sc_lg	NK.cells.rest_lg																			2	2.08E-02	2.08E-02
73	NK.cells.rest_lg	Macrophages_sc_lg											2.08E-02	-	2.08E-02	-							
74	Epithelial	pM2																			0	1.00E+00	1.00E+00
74	pM2	Epithelial																					
75	Endothelial.cells_lg	pM0		-																	3	1.27E-04	1.27E-04
75	pM0	Endothelial.cells_lg		-									1.27E-04	-					1.27E-04	-			
76	CCN4	NK.cells.rest_lg																			0	1.00E+00	1.00E+00
76	NK.cells.rest_lg	CCN4																					
77	NK.cells.active_lg	T.cells.CD8_lg	+	+	3.93E-03	+	1.05E-04	+	1.05E-04	+			1.19E-03	+	1.19E-03	+	3.93E-03	+	3.93E-03	+	9	1.05E-04	3.93E-03
77	T.cells.CD8_lg	NK.cells.active_lg	+	+																			
78	CAF_lg	CD4Tcell_sc_lg																			0	1.00E+00	1.00E+00
78	CD4Tcell_sc_lg	CAF_lg																					
79	CAF_lg	CCN4																			1	2.35E-01	2.35E-01
79	CCN4	CAF_lg													2.35E-01	+							
80	Endothelial.cells_lg	Epithelial													9.69E-04	+					1	9.69E-04	9.69E-04
80	Epithelial	Endothelial.cells_lg																					
81	Macrophages_sc_lg	NK.cells.active_lg																			0	1.00E+00	1.00E+00
81	NK.cells.active_lg	Macrophages_sc_lg																					
82	Macrophages_sc_lg	pM1											5.29E-07	+	5.29E-07	+					2	5.29E-07	5.29E-07
82	pM1	Macrophages_sc_lg																					
83	Cancer	NK.cells.active_lg					8.85E-16	+	8.85E-16	+			8.85E-16	+	8.85E-16	+					4	8.85E-16	8.85E-16
83	NK.cells.active_lg	Cancer																					
84	Epithelial	pM0											3.92E-05	-	3.92E-05	-					2	3.92E-05	3.92E-05
84	pM0	Epithelial																					

Supplementary Table 3. List of edges and corresponding arcs, whether an arc was predicted to promote (+) or inhibit (-) the target node, and the strength inferred using the different structure learning algorithms in analyzing the features present in primary melanoma tissue samples in the TCGA SKCM dataset. Rows highlighted in blue indicate arcs that were included in the consensus seed network (a.k.a. “whitelist"), yellow indicate that the directionality was unclear, and red indicate arcs included in the “blacklist". The edge numbers correspond to the x-axis in Supplementary Figure 3, panel B.

Edge No	from	to	mmpc CorSign	hiton CorSign	pc_stable strength	pcstable CorSign	gs strength	gs CorSign	iamb strength	iamb CorSign	iambfdr strength	iambfdr CorSign	tabu strength	tabu CorSign	hc strength	hc CorSign	mmhc strength	mmhc CorSign	rsmax2 strength	rsmax2 CorSign	Hit Count	Min strength	Max strength
1	CCN4	Mesenchymal	+	+	3.83E-06	+			1.14E-06	+											4	1.14E-06	3.83E-06
1	Mesenchymal	CCN4	+	+																			
2	pM0	pM1	-	-											9.96E-07	-	9.96E-07	-	9.96E-07	-	9	1.59E-34	9.96E-07
2	pM1	pM0	-	-	1.59E-34	-	1.59E-34	-	1.59E-34	-	1.59E-34	-											
3	pM0	pM2	-	-											6.36E-46	-	2.04E-18	-	2.04E-18	-	9	6.36E-46	2.04E-18
3	pM2	pM0	-	-	6.36E-46	-	6.36E-46	-	6.36E-46	-	6.36E-46	-											
4	CAF_lg	Mesenchymal	+	+											3.26E-18	+					9	3.26E-18	3.26E-18
4	Mesenchymal	CAF_lg	+	+	3.26E-18	+	3.26E-18	+	3.26E-18	+	3.26E-18	+					3.26E-18	+	3.26E-18	+			
5	Cancer	CCN4																			0	1.00E+00	1.00E+00
5	CCN4	Cancer																					
6	NK.cells.active_lg	NK.cells.rest_lg	-	-	2.37E-09	-									2.37E-09	-					9	2.01E-10	2.37E-09
6	NK.cells.rest_lg	NK.cells.active_lg	-	-			2.01E-10	-	2.01E-10	-	2.01E-10	-					2.01E-10	-	2.01E-10	-			
7	B.cells.ive_lg	T.cells.CD8_lg																			0	1.00E+00	1.00E+00
7	T.cells.CD8_lg	B.cells.ive_lg																					
8	CAF_lg	proliferation																			0	1.00E+00	1.00E+00
8	proliferation	CAF_lg																					
9	Endothelial.cells_lg	proliferation																			0	1.00E+00	1.00E+00
9	proliferation	Endothelial.cells_lg																					
10	Cancer	Epithelial																			0	1.00E+00	1.00E+00
10	Epithelial	Cancer																					
11	Cancer	proliferation																			0	1.00E+00	1.00E+00
11	proliferation	Cancer																					
12	CD4Tcell_sc_lg	Macrophages_sc_lg	-	-																	8	1.96E-06	2.61E-04
12	Macrophages_sc_lg	CD4Tcell_sc_lg	-	-	2.61E-04	-	2.61E-04	-	2.61E-04	-					1.96E-06	-	2.61E-04	-	2.61E-04	-			
13	pM1	T.cells.CD8_lg																			0	1.00E+00	1.00E+00
13	T.cells.CD8_lg	pM1																					
14	Macrophages_sc_lg	T.cells.CD8_lg	+	+	2.11E-05	+	1.43E-07	+	1.43E-07	+	1.43E-07	+			1.23E-11	+	1.43E-07	+	2.11E-05	+	9	1.23E-11	2.11E-05
14	T.cells.CD8_lg	Macrophages_sc_lg	+	+																			
15	CD4Tcell_sc_lg	Endothelial.cells_lg																			0	1.00E+00	1.00E+00
15	Endothelial.cells_lg	CD4Tcell_sc_lg																					
16	CAF_lg	T.cells.CD8_lg													1.58E-05	-					1	1.58E-05	1.58E-05
16	T.cells.CD8_lg	CAF_lg																					
17	B.cells.ive_lg	CD4Tcell_sc_lg																			0	1.00E+00	1.00E+00
17	CD4Tcell_sc_lg	B.cells.ive_lg																					
18	Epithelial	Neutrophils_lg																			0	1.00E+00	1.00E+00
18	Neutrophils_lg	Epithelial																					
19	Cancer	Endothelial.cells_lg																			0	1.00E+00	1.00E+00
19	Endothelial.cells_lg	Cancer																					
20	B.cells.ive_lg	Endothelial.cells_lg																			0	1.00E+00	1.00E+00
20	Endothelial.cells_lg	B.cells.ive_lg																					
21	Cancer	pM2																			0	1.00E+00	1.00E+00
21	pM2	Cancer																					
22	Cancer	Mesenchymal																			0	1.00E+00	1.00E+00
22	Mesenchymal	Cancer																					
23	pM2	proliferation																			0	1.00E+00	1.00E+00
23	proliferation	pM2																					
24	Neutrophils_lg	pM2																			0	1.00E+00	1.00E+00
24	pM2	Neutrophils_lg																					
25	Macrophages_sc_lg	Neutrophils_lg																			0	1.00E+00	1.00E+00
25	Neutrophils_lg	Macrophages_sc_lg																					
26	CAF_lg	pM1																			0	1.00E+00	1.00E+00
26	pM1	CAF_lg																					
27	pM1	pM2													9.70E-30	-					1	9.70E-30	9.70E-30
27	pM2	pM1																					
28	NK.cells.rest_lg	T.cells.CD8_lg																			0	1.00E+00	1.00E+00
28	T.cells.CD8_lg	NK.cells.rest_lg																					
29	CD4Tcell_sc_lg	NK.cells.active_lg																			0	1.00E+00	1.00E+00
29	NK.cells.active_lg	CD4Tcell_sc_lg																					
30	CD4Tcell_sc_lg	pM1																			0	1.00E+00	1.00E+00
30	pM1	CD4Tcell_sc_lg																					
31	Cancer	pM1																			0	1.00E+00	1.00E+00
31	pM1	Cancer																					
32	Cancer	pM0																			0	1.00E+00	1.00E+00
32	pM0	Cancer																					
33	Endothelial.cells_lg	Mesenchymal	+	+	1.28E-07	+															8	3.73E-08	1.28E-07
33	Mesenchymal	Endothelial.cells_lg	+	+			3.73E-08	+	3.73E-08	+					3.73E-08	+	3.73E-08	+	3.73E-08	+			
34	B.cells.ive_lg	pM1																			0	1.00E+00	1.00E+00
34	pM1	B.cells.ive_lg																					
35	CAF_lg	Macrophages_sc_lg	+	+	1.45E-08	+	1.45E-08	+	1.45E-08	+	1.45E-08	+			1.02E-11	+	1.45E-08	+	1.45E-08	+	9	1.02E-11	1.45E-08
35	Macrophages_sc_lg	CAF_lg	+	+																			
36	B.cells.ive_lg	Cancer																			0	1.00E+00	1.00E+00
36	Cancer	B.cells.ive_lg																					
37	CAF_lg	pM2																			0	1.00E+00	1.00E+00
37	pM2	CAF_lg																					
38	Mesenchymal	pM0																			0	1.00E+00	1.00E+00
38	pM0	Mesenchymal																					
39	Endothelial.cells_lg	pM1																			0	1.00E+00	1.00E+00
39	pM1	Endothelial.cells_lg																					
40	CCN4	proliferation																			0	1.00E+00	1.00E+00
40	proliferation	CCN4																					
41	Cancer	T.cells.CD8_lg																			0	1.00E+00	1.00E+00
41	T.cells.CD8_lg	Cancer																					
42	Macrophages_sc_lg	pM0																			0	1.00E+00	1.00E+00
42	pM0	Macrophages_sc_lg																					
43	NK.cells.rest_lg	pM0																			0	1.00E+00	1.00E+00
43	pM0	NK.cells.rest_lg																					
44	Mesenchymal	Neutrophils_lg																			0	1.00E+00	1.00E+00
44	Neutrophils_lg	Mesenchymal																					
45	CCN4	Macrophages_sc_lg																			0	1.00E+00	1.00E+00
45	Macrophages_sc_lg	CCN4																					
46	Cancer	NK.cells.rest_lg																			0	1.00E+00	1.00E+00
46	NK.cells.rest_lg	Cancer																					
47	CD4Tcell_sc_lg	Epithelial																			1	1.36E-02	1.36E-02
47	Epithelial	CD4Tcell_sc_lg													1.36E-02	+							
48	B.cells.ive_lg	NK.cells.rest_lg																			0	1.00E+00	1.00E+00
48	NK.cells.rest_lg	B.cells.ive_lg																					
49	Epithelial	Mesenchymal																			0	1.00E+00	1.00E+00
49	Mesenchymal	Epithelial																					
50	Endothelial.cells_lg	Neutrophils_lg																			0	1.00E+00	1.00E+00
50	Neutrophils_lg	Endothelial.cells_lg																					
51	Macrophages_sc_lg	proliferation													1.72E-04	-					1	1.72E-04	1.72E-04
51	proliferation	Macrophages_sc_lg																					
52	Mesenchymal	T.cells.CD8_lg																			0	1.00E+00	1.00E+00
52	T.cells.CD8_lg	Mesenchymal																					
53	CCN4	NK.cells.active_lg																			0	1.00E+00	1.00E+00
53	NK.cells.active_lg	CCN4																					
54	CAF_lg	Epithelial																			0	1.00E+00	1.00E+00
54	Epithelial	CAF_lg																					
55	B.cells.ive_lg	CAF_lg																			0	1.00E+00	1.00E+00
55	CAf_lg	B.cells.ive_lg																					
56	NK.cells.rest_lg	proliferation																			0	1.00E+00	1.00E+00
56	proliferation	NK.cells.rest_lg																					
57	B.cells.ive_lg	Epithelial																			0	1.00E+00	1.00E+00
57	Epithelial	B.cells.ive_lg																					
58	B.cells.ive_lg	CCN4																			0	1.00E+00	1.00E+00
58	CCN4	B.cells.ive_lg																					
59	Cancer	Macrophages_sc_lg																			0	1.00E+00	1.00E+00
59	Macrophages_sc_lg	Cancer																					
60	CAF_lg	Endothelial.cells_lg																			0	1.00E+00	1.00E+00
60	Endothelial.cells_lg	CAF_lg																					
61	CCN4	pM0																			0	1.00E+00	1.00E+00
61	pM0	CCN4																					
62	Endothelial.cells_lg	T.cells.CD8_lg																			0	1.00E+00	1.00E+00
62	T.cells.CD8_lg	Endothelial.cells_lg																					
63	Macrophages_sc_lg	Mesenchymal																			0	1.00E+00	1.00E+00
63	Mesenchymal	Macrophages_sc_lg																					
64	B.cells.ive_lg	Macrophages_sc_lg																			0	1.00E+00	1.00E+00
64	Macrophages_sc_lg	B.cells.ive_lg																					
65	Mesenchymal	pM2																			0	1.00E+00	1.00E+00
65	pM2	Mesenchymal																					
66	NK.cells.active_lg	pM0													3.16E-05	-					1	3.16E-05	3.16E-05
66	pM0	NK.cells.active_lg																					
67	B.cells.ive_lg	Mesenchymal																			0	1.00E+00	1.00E+00
67	Mesenchymal	B.cells.ive_lg																					
68	CCN4	Endothelial.cells_lg																			0	1.00E+00	1.00E+00
68	Endothelial.cells_lg	CCN4																					
69	CAF_lg	Cancer																			0	1.00E+00	1.00E+00
69	Cancer	CAF_lg																					
70	Neutrophils_lg	pM0																			0	1.00E+00	1.00E+00
70	pM0	Neutrophils_lg																					
71	CCN4	Neutrophils_lg																			0	1.00E+00	1.00E+00
71	Neutrophils_lg	CCN4																					
72	Neutrophils_lg	pM1																			0	1.00E+00	1.00E+00
72	pM1	Neutrophils_lg																					
73	Macrophages_sc_lg	NK.cells.rest_lg																			0	1.00E+00	1.00E+00
73	NK.cells.rest_lg	Macrophages_sc_lg																					
74	Epithelial	pM2																			0	1.00E+00	1.00E+00
74	pM2	Epithelial																					
75	Endothelial.cells_lg	pM0																			0	1.00E+00	1.00E+00
75	pM0	Endothelial.cells_lg																					
76	CCN4	NK.cells.rest_lg	+	+	1.57E-02	+	1.32E-03	+	1.32E-03	+					1.57E-02	+	1.32E-03	+	1.32E-03	+	8	1.32E-03	1.57E-02
76	NK.cells.rest_lg	CCN4	+	+																			
77	NK.cells.active_lg	T.cells.CD8_lg		+	1.87E-03	+													1.87E-03	+	3	1.87E-03	1.87E-03
77	T.cells.CD8_lg	NK.cells.active_lg		+																			
78	CAF_lg	CD4Tcell_sc_lg													5.54E-03	+					1	5.54E-03	5.54E-03
78	CD4Tcell_sc_lg	CAF_lg																					
79	CAF_lg	CCN4																			1	4.75E-06	4.75E-06
79	CCN4	CAF_lg													4.75E-06	+							
80	Endothelial.cells_lg	Epithelial													1.01E-02	+					1	1.01E-02	1.01E-02
80	Epithelial	Endothelial.cells_lg																					
81	Macrophages_sc_lg	NK.cells.active_lg																			1	1.29E-04	1.29E-04
81	NK.cells.active_lg	Macrophages_sc_lg													1.29E-04	+							
82	Macrophages_sc_lg	pM1																			1	1.64E-02	1.64E-02
82	pM1	Macrophages_sc_lg													1.64E-02	+							
83	Cancer	NK.cells.active_lg																			0	1.00E+00	1.00E+00
83	NK.cells.active_lg	Cancer																					
84	Epithelial	pM0																			0	1.00E+00	1.00E+00

Supplementary Table 4. Proliferation metrics associated $CD4^+$ and $CD8^+$ T cells stimulated in vitro in different conditions. Dil: fraction diluted; PF: Precursor frequency, %dividing cells; PI: Proliferation index; and SD^D: proliferation variance. Summary statistics were calculated from three biological replicates and represented as mean (standard deviation). Statistical significance was assessed using type III repeated measures ANOVA and was two-sided, where * indicates a p-value < 0.05.

	Live CD4+ T cells				Live CD8+ T cells			
Experimental Conditions	Dil	PF	PI	SD^D	Dil	PF	PI	SD^D
AP beads + rCCN4	0.670 (0.012)	0.392 (0.014)	1.407 (0.033)	0.274	0.983 (0.003)	0.851 (0.014)	2.655 (0.045)	0.103
AP beads + CCN4 KO TCM	0.472* (0.008)	0.221* (0.003)	1.404 (0.023)	0.274	0.914* (0.015)	0.715* (0.032)	1.729* (0.044)	0.205
AP beads + WT TCM	0.552* (0.047)	0.282* (0.038)	1.403 (0.031)	0.272	0.920* (0.020)	0.704* (0.044)	1.923* (0.052)	0.189
AP beads	0.655 (0.043)	0.366 (0.035)	1.473 (0.060)	0.255	0.982 (0.002)	0.841 (0.026)	2.756 (0.187)	0.106
No stimulation	0.046 (0.025)	0.016 (0.013)	1.763 (1.056)	0.371	0.062 (0.018)	0.008 (0.004)	2.520 (0.441)	0.229

Supplementary Table 5. List of fluorophore-conjugated anti-mouse antibodies using to quantify cell subsets by flow cytometry.

Marker	Clone	Fluorophore	Dilution	Manufacturer
LIVE/DEAD Fix	--	Violet/Pacific Blue	--	Invitrogen
CD45	30-F11	BB515	1:80	BD Biosciences #564590
CD3e	500A2	Alexa Fluor 700	1:50	BioLegend #152316
CD4	GK1.5	APC-Cy7	1:80	BD Biosciences #552051
CD8a	REA601	APC	1:50	Miltenyi 130-109-248
CD161 (NK-1.1)	PK136	APC-Cy7	1:20	BioLegend #108723
CD45R/B220	RA3-6B2	APC	1:80	BioLegend #103212
CD49b	DX5	PerCP/Cy5.5	1:80	Biolegend #108915
CD11b	M1/70	PerCP/Cy5.5	1:80	eBioscience #45-0112-80
CD11c	N418	PE	1:40	eBioscience #12-0114-81
F4/80	BM8	APC-Cy7	1:20	BioLegend #123117
Ly-6G/Ly-6C (Gr-1)	RB6-8C5	APC	1:80	BioLegend #108412
CD279 (PD-1)	REA802	PE	1:20	BioLegend #135205
I-A/I-E (MHC-II)	M5/114.15.2	Alexa Fluor 700	1:200	BioLegend #107622

Supplementary Table 6. List of primers used for RT-PCR.

Oligo Name	Sequence (5' to 3')
qMmSnai1-F	CACACGCTGCCTTGTGTCT
qMmSnai1-R	GGTCAGCAAAAGCACGGTT
qMmSnai2-F	CTCACCTCGGGAGCATACAG
qMmSnai2-R	GACTTACACGCCCCAAGGATG
qH/MGapdh-F	TGCACCACCAACTGCTTAGC
qH/MGapdh-R	GGCATGGACTGTGGTCATGAG
qMmZeb1-F	ACCCCTTCAAGAACCGCTTT
qMmZeb1-R	CAATTGGCCACCACTGCTAA
qMmZeb2-F	AGGCATATGGTGACGCACAA
qMmZeb2-R	CTTGAACTTGCGGTTACCTGC
qMmCdh1-F	CAGCCTTCTTTTCGGAAGACT
qMmCdh1-R	GGTAGACAGCTCCCTATGACTG
qMmCdh2-F	CCAGCAGATTTCAAGGTGGAC
qMmCdh2-R	TTACAGCTACCTGCCACTTTTC
qMmFn1-F	ATGTGGACCCCTCCTGATAGT
qMmFn1-R	GCCCAGTGATTTCAGCAAAGG

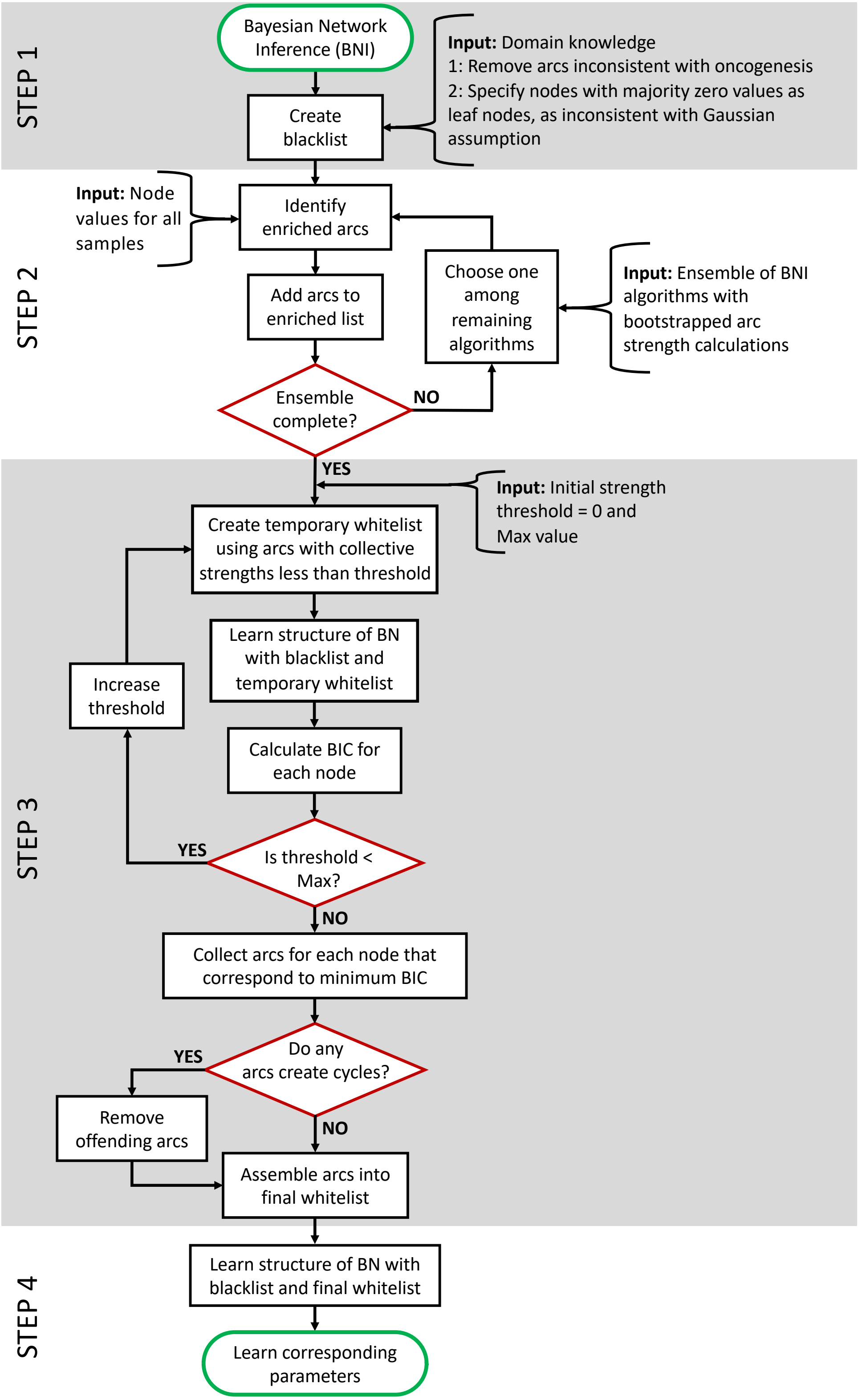

Supplementary Figure 1. Analysis workflow for Bayesian Network Inference. The causal structure associated with cell-level networks were inferred from data using a four-step process. The four steps corresponded to specifying a “blacklist" based on prior information; generating an ensemble of potential arcs using 10 different structural learning algorithms, that is the consensus of edges contained in the seed network; filtering potential arcs based on a trade-off between regression accuracy and model complexity as quantified by the Bayesian Information Criterion (BIC) to create a “whitelist"; and learning the network structure and the corresponding parameter values using both the “blacklist" and “whitelist".

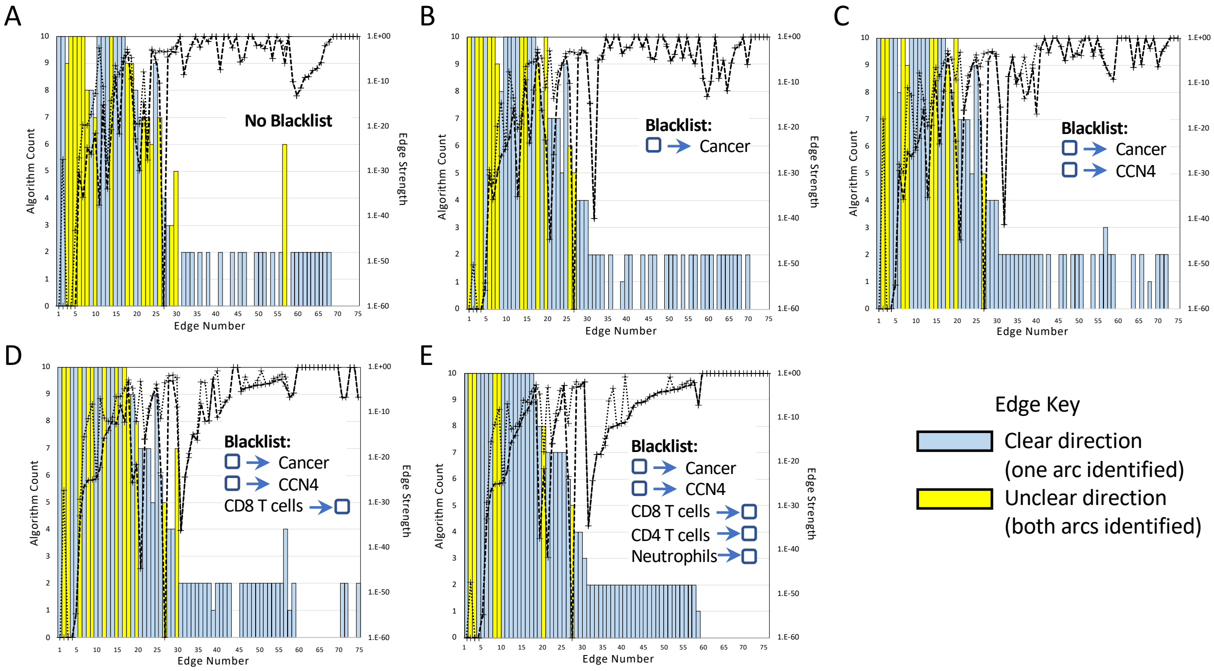

Supplementary Figure 2. Impact of arcs included in blacklist on consensus edges in the seed network. Using the TCGA BRCA dataset, a consensus seed network was generated multiple times using a blacklist that was progressively increased from an empty list (A) to a list that included cancer (B-E) and CCN4 (C-E) as tail nodes and CD8 T cells (D,E) and CD4 T cells and neutrophils (E) as head nodes. As the number of arcs included in the blacklist was increased, the number of edges with unclear direction was decreased. Seventeen edges had an unclear direction without specifying a blacklist (A), while only 6 edges had an unclear direction in the final blacklist (E). Edges were ordered based on the number of algorithms that detected either arc were enriched (bar graph - left axis) and the strength of enrichment (dotted lines - right axis). The lines associated with the strength of enrichment represent the minimum (dashed line) and maximum (dotted line) values obtained by the different algorithms for each edge. Coloring of bar graph indicates whether the direction associated with an edge was clear (gray - i.e., only one arc was found to be significant among the algorithms) or unclear (yellow - i.e., both arcs were found to be significant among the algorithms).

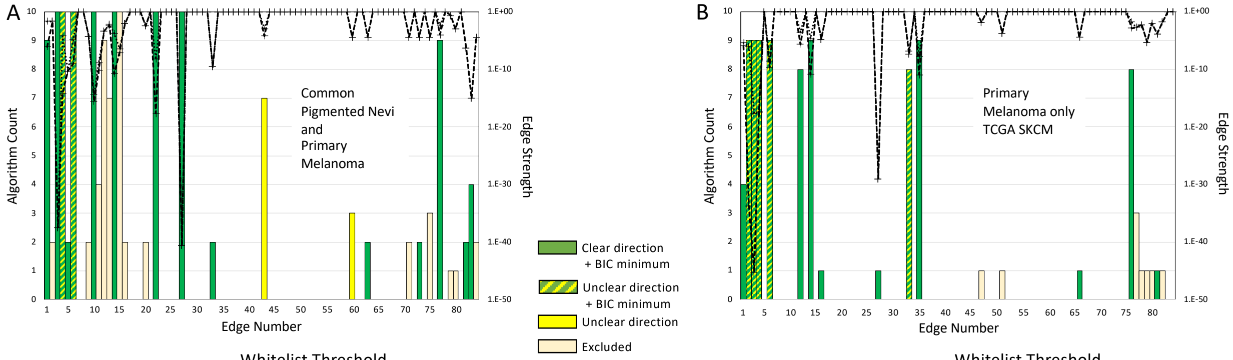

A — Whitelist Threshold

Node	Empty Graph	< 0	< 1E-20	< 1E-15	< 1E-10	< 1E-05	< 1E-03	All
	BIC / Number of Parental Arcs							
Cancer	**35.3 / 0**	35.3 / 0	35.3 / 0	35.3 / 0	35.3 / 0	35.3 / 0	35.3 / 0	35.3 / 0
CCN4	15.6 / 0	15.6 / 0	15.6 / 0	15.6 / 0	**12.9 / 1**	12.9 / 1	12.9 / 1	12.9 / 1
B Cells naive	**17.4 / 0**	17.4/ 0	17.4 / 0	17.4 / 0	17.4 / 0	17.4 / 0	17.4 / 0	18.7 / 1
CAF	11.5 / 0	**10.0 / 1**	10.0 / 1	10.0 / 1	10.0 / 1	10.0 / 1	10.0 / 1	11.9 / 2
CD4 T cells	**15.4 / 0**	15.8 / 2	15.8 / 2	15.8 / 2	15.8 / 2	15.8 / 2	15.8 / 2	15.8 / 2
CD8 T cells	22.3 / 0	18.5 / 3	**17.0 / 2**	17.0 / 2	17.0 / 2	17.0 / 2	17.0 / 2	19.6 / 4
Endothelial cells	**16.8 / 0**	16.8 / 0	16.8 / 0	16.8 / 0	16.8 / 0	16.8 / 0	16.8 / 0	16.8 / 0
Epithelial	14.8 / 0	**10.0 / 1**	10.0 / 1	10.0 / 1	10.0 / 1	10.0 / 1	11.9 / 2	11.9 / 2
Macrophages	19.2 / 0	19.2 / 0	19.2 / 0	19.2 / 0	19.2 / 0	19.2 / 0	**17.6 / 2**	17.6 / 2
Mesenchymal	19.5 / 0	13.4 / 2	13.4 / 2	13.4 / 2	13.4 / 2	**13.1 / 3**	13.1 / 3	13.1 / 3
Neutrophils	**19.2 / 0**	19.2 / 0	19.2 / 0	19.2 / 0	19.2 / 0	19.2 / 0	19.6 / 1	19.6 / 1
NK cells active	17.8 / 0	17.8 / 0	17.8 / 1	**13.8 / 2**	13.8 / 2	13.8 / 2	13.8 / 2	13.8 / 2
NK cells resting	**13.1 / 0**	15.9 / 2	13.1 / 0	13.1 / 0	13.1 / 0	13.1 / 0	13.1 / 0	13.1 / 0
pM0	**11.2 / 0**	11.2 / 0	11.2 / 0	11.2 / 0	11.2 / 0	11.2 / 0	11.4 / 1	11.4 / 1
pM1	19.2 / 0	19.2 / 0	19.2 / 0	19.2 / 0	19.2 / 0	**16.3 / 1**	16.3 / 1	18.4 / 2
pM2	21.6 / 0	**7.7 / 2**	7.7 / 2	7.7 / 2	7.7 / 2	7.7 / 2	7.7 / 2	7.7 / 2
Proliferation	**16.8 / 0**	16.8 / 0	16.8 / 0	16.8 / 0	16.8 / 1	16.8 / 1	17.0 / 2	17.0 / 2

B — Whitelist Threshold

Node	Empty Graph	< 0	< 1E-20	< 1E-15	< 1E-10	< 1E-05	< 1E-03	All
	BIC / Number of Parental Arcs							
Cancer	**0.0 / 0**	0.0 / 0	0.0 / 0	0.0 / 0	0.0 / 0	0.0 / 0	0.0 / 0	0.0 / 0
CCN4	**10.9 / 0**	10.9 / 0	10.9 / 0	10.9 / 0	10.9 / 0	10.9 / 0	10.9 / 0	10.9 / 0
B Cells naive	**28.5 / 0**	28.5/ 0	28.5 / 0	28.5 / 0	28.5 / 0	28.5 / 0	28.5 / 0	28.5 / 0
CAF	20.2 / 0	**15.8 / 1**	15.8 / 1	15.8 / 1	15.8 / 1	20.9 / 1	20.9 / 1	20.9 / 1
CD4 T cells	35.9 / 0	**32.3 / 1**	32.3 / 1	32.3 / 1	32.3 / 1	32.3 / 1	32.3 / 1	33.6 / 3
CD8 T cells	20.8 / 0	**18.9 / 1**	18.9 / 1	18.9 / 1	18.9 / 1	18.9 / 1	19.5 / 2	21.4 / 3
Endothelial cells	25.0 / 0	**22.8 / 1**	22.8 / 1	22.8 / 1	22.8 / 1	22.8 / 1	22.8 / 1	25.0 / 0
Epithelial	**16.0 / 0**	16.0 / 0	16.0 / 0	16.0 / 0	16.0 / 0	16.0 / 0	16.0 / 0	18.0 / 1
Macrophages	22.2 / 0	19.6 / 1	19.6 / 1	19.6 / 1	19.6 / 1	19.6 / 1	**19.0 / 2**	20.4 / 3
Mesenchymal	20.3 / 0	20.3 / 0	20.3 / 0	20.3 / 0	20.3 / 0	**16.6 / 2**	16.6 / 2	16.6 / 2
Neutrophils	**25.6 / 0**	25.6 / 0	25.6 / 0	25.6 / 0	25.6 / 0	25.6 / 0	25.6 / 0	25.6 / 0
NK cells active	32.4 / 0	**25.5 / 1**	25.5 / 1	25.5 / 1	25.5 / 1	25.5 / 1	25.5 / 1	25.5 / 1
NK cells resting	33.2 / 0	**31.9 / 1**	31.9 / 1	31.9 / 1	31.9 / 1	31.9 / 1	33.2 / 0	31.9 / 1
pM0	23.9 / 0	23.9 / 0	23.9 / 0	23.9 / 0	23.9 / 0	23.9 / 0	**23.6 / 1**	23.6 / 1
pM1	19.1 / 0	**17.6 / 1**	17.6 / 1	17.6 / 1	17.6 / 1	17.6 / 1	17.6 / 1	17.6 / 1
pM2	20.9 / 0	15.0 / 1	**10.8 / 2**	10.8 / 2	10.8 / 2	10.8 / 2	10.8 / 2	10.8 / 2
Proliferation	**16.8 / 0**	16.8 / 0	16.8 / 0	16.8 / 0	16.8 / 0	16.8 / 0	17.0 / 1	17.0 / 1

Supplementary Figure 3. Summary of the evidence obtained from two melanoma datasets supporting the consensus edges in the seed network. Analysis of datasets containing samples from both common pigmented nevi and primary melanoma (A) and from only primary melanoma (B). For each edge listed in Supplementary Tables 2 and 3, the number of algorithms that detected than an edge was enriched (bar graph - left axis) and the strength of enrichment (dotted lines - right axis) are shown. The lines associated with the strength of enrichment represent the minimum (dashed line) and maximum (dotted line) values obtained by the different algorithms for each edge. Bar graph coloring indicates whether an edge was significantly enriched with a clear direction and included in the set of arcs associated with the BIC minimum (green), significantly enriched without a clear direction but included in the set of arcs associated with the BIC minimum (yellow/green), significantly enriched but without a clear direction (yellow), or excluded from the consensus seed network list (tan).

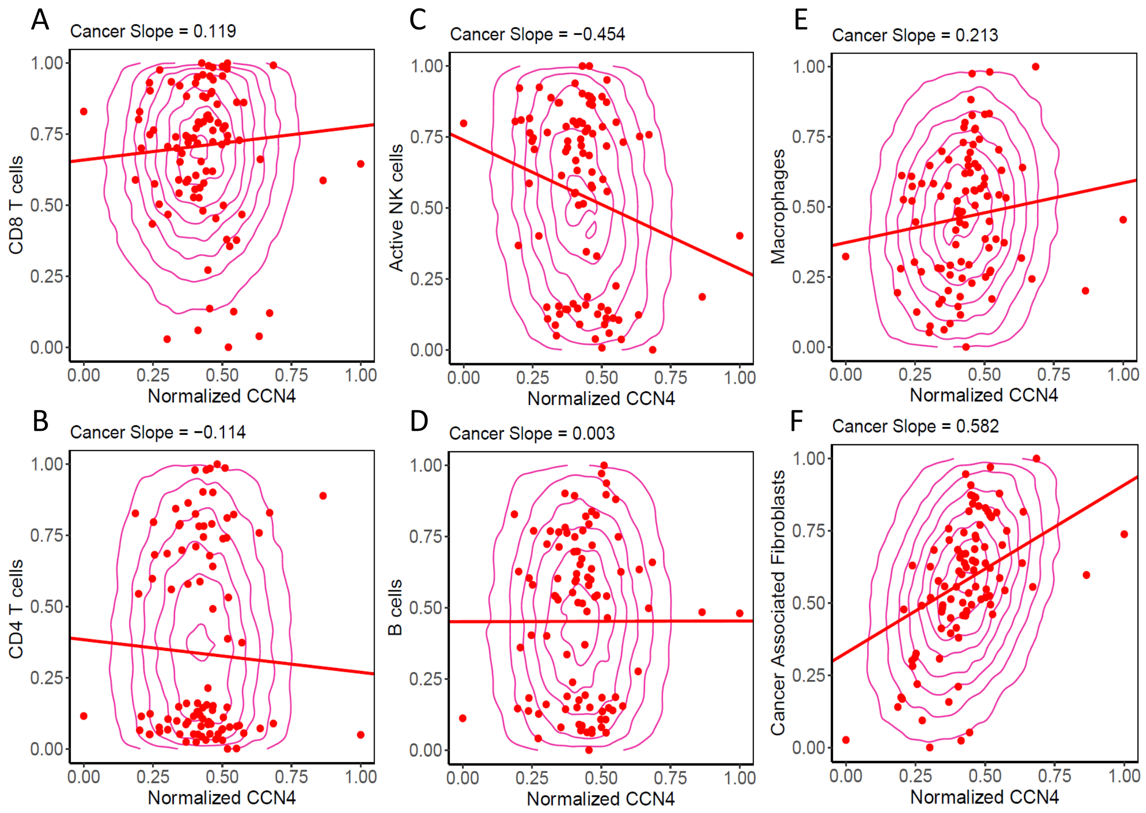

Supplementary Figure 4. Conditional probability query of the SKCM DAG compared against digital cytometry estimates obtained from experimental data. Experimental samples obtained from primary melanoma tissue are shown as red dots. Samples of the conditional probability model for Cancer > 0.95 (red contours) for CD8 T cells (A), CD4 T cells (B), active NK cells (C), B cells (D), Macrophages (E) and Cancer Associated Fibroblasts (F). Linear trend lines are superimposed on the conditional probability samples.

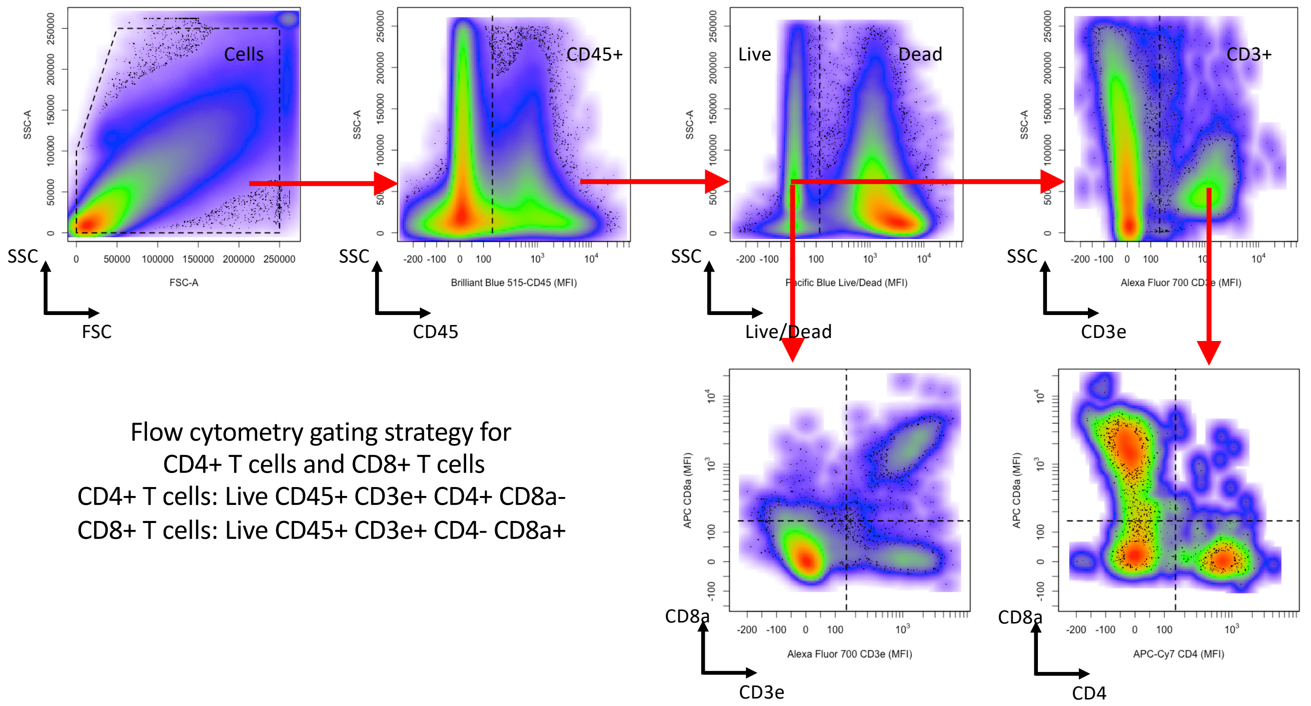

Supplementary Figure 5. Flow cytometry gating strategy for T cells. CD45 staining versus side scatter area was used to gate for CD45+ cells. Live Dead Pacific Blue staining versus side scatter area was used to then gate for Live CD45$^+$ cells, which were then gated based on CD3e$^+$ expression. Live CD45$^+$ CD3e$^+$ cells were further subdivided into CD8$^+$ T cells (live CD8$^+$ CD3e$^+$ CD45$^+$ cells), CD4 T cells (live CD4$^+$ CD3e$^+$ CD45$^+$ cells), and double negative T cells (live CD8$^-$ CD4$^-$ CD3e$^+$ CD45$^+$ cells).

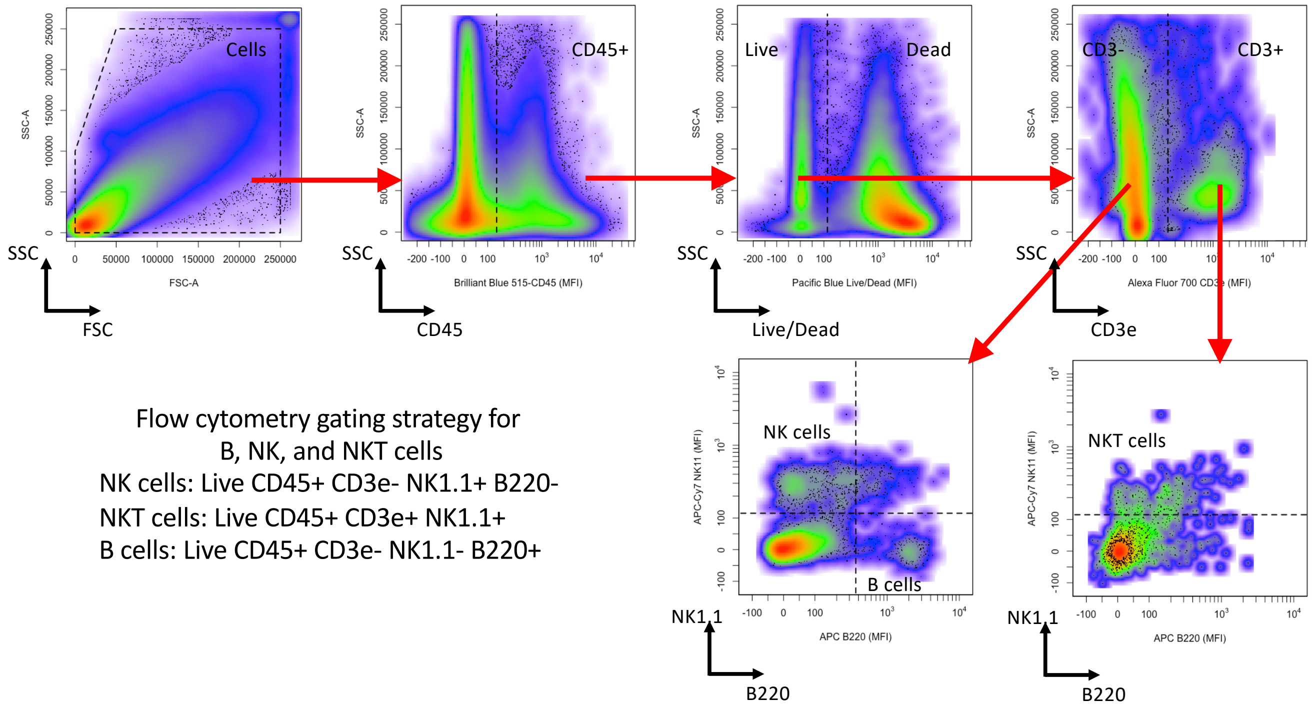

Supplementary Figure 6. Flow cytometry gating strategy for B, NK, and NKT cells. CD45 staining versus side scatter area was used to gate for CD45+ cells. Live Dead Pacific Blue staining versus side scatter area was used to gate for Live CD45$^+$ cells, which were then subdivided into B cells (live NK1.1$^-$ B220$^+$ CD3$^-$ CD45$^+$ cells), NK cells (live NK1.1$^+$ B220$^-$ CD3$^-$ CD45$^+$ cells), and NKT cells (live NK1.1$^+$ CD3e$^+$ CD45$^+$ cells).

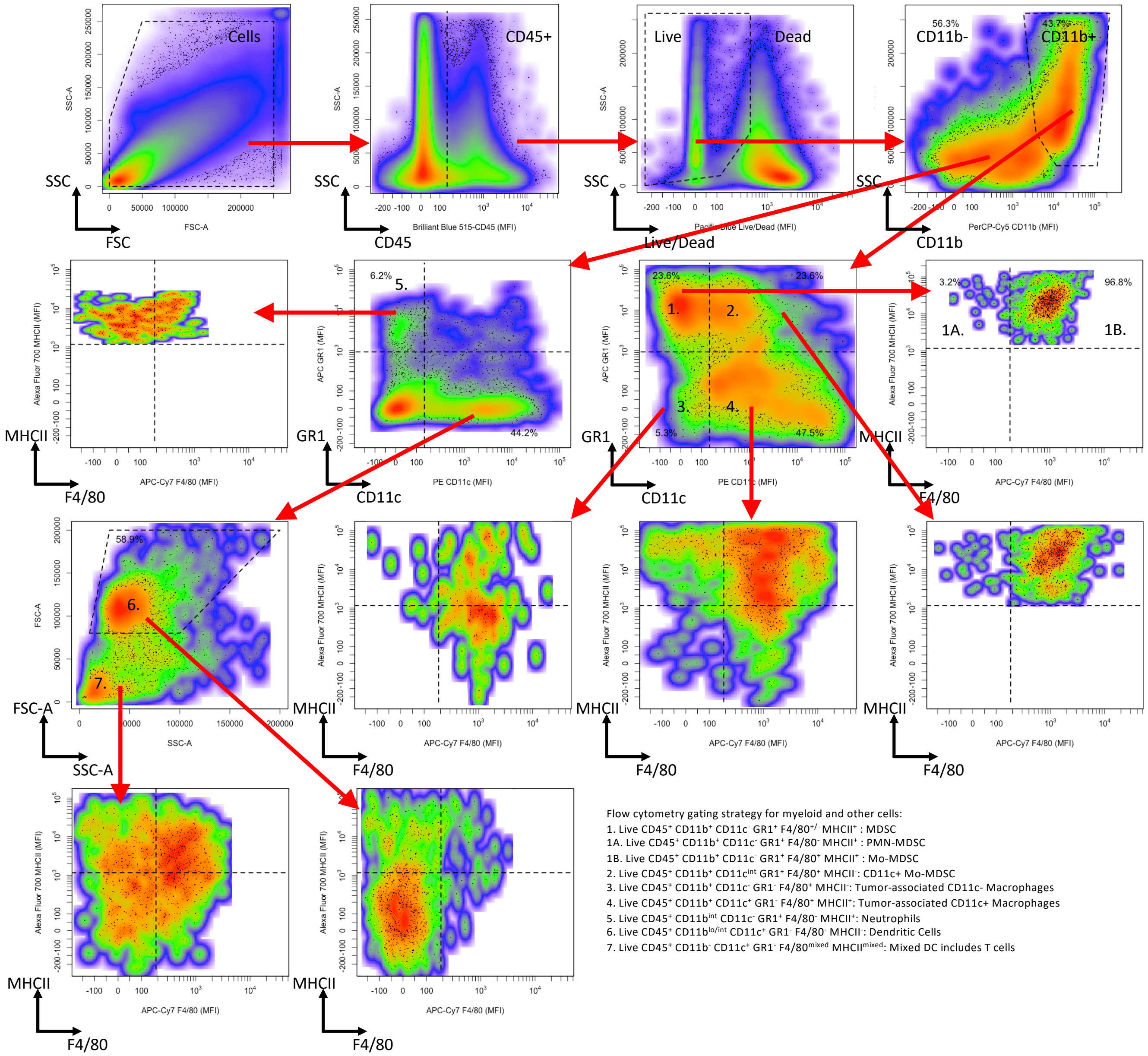

Supplementary Figure 7. Flow cytometry gating strategy for Tumor associated neutrophils and myeloid cell subsets. CD45 staining versus side scatter area was used to gate for CD45^{+} cells. Live Dead Pacific Blue staining versus side scatter area was used to gate for Live CD45^{+} cells, which were then subdivided into subsets based on CD11b staining followed by Gr1 versus CD11c staining. From the CD11b^{+} gate, myeloid-derived suppressor cells (MDSC) (live CD45^{+} CD11b^{+} Gr1^{+} cells) were subdivided into CD11c$^{int/+}$ MDSC (F4/80^{+} MHCII^{+}) and CD11c^{-} MDSC (F4/80^{mixed} MHCII^{+}). Also from the CD11b^{+} gate, macrophages (live Gr1^{-} F4/80^{+} CD11b^{+} CD45^{+} cells) were subdivided into tumor-associated CD11c^{+} (CD11c$^{int/+}$ MHCII^{hi}) and CD11c^{-} (CD11c^{-} MHCII^{lo})subsets. The CD11b^{-} subset included tumor-associated neutrophils (TAN) (Gr1^{+} CD11c^{-} CD11b^{int} MHCII^{hi} F4/80^{-}) and dendritic cells (Gr1^{-} CD11c^{+} CD11b$^{lo/int}$ FSC-A^{hi} MHCII^{lo} F4/80^{-}).

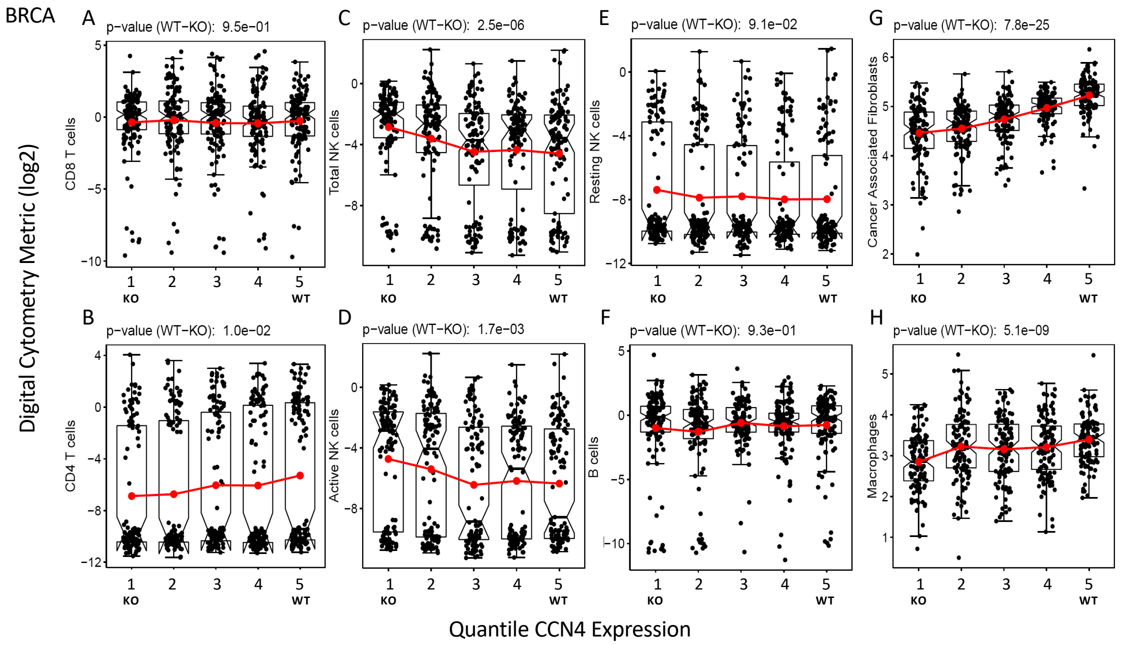

Supplementary Figure 8. Distribution in digital cytometry features extracted from the BRCA dataset stratified into quantiles based on CCN4 expression. The mean value for quantile 1 was used to represent CCN4 KO phenotype while quantile 5 was used to represent WT phenotype. A two-sided Wilcoxon test was used to assess significance. Summary quantile statistics from n = 582 biologically independent samples are represented by the box-whisker plot, which include: upper whisker = the minimum of either the 75th percentile plus 1.5 times the inter-quartile range or the maximum value; upper box = 75th percentile; upper edge of notch = median + 1.57 times the inter-quartile range divided by the square root of n; cross bar = median; lower edge of notch = median - 1.57 times the inter-quartile range divided by the square root of n; lower box = 25th percentile; and lower whisker = the maximum of either the 25th percentile minus 1.5 times the inter-quartile range or the minimum value.

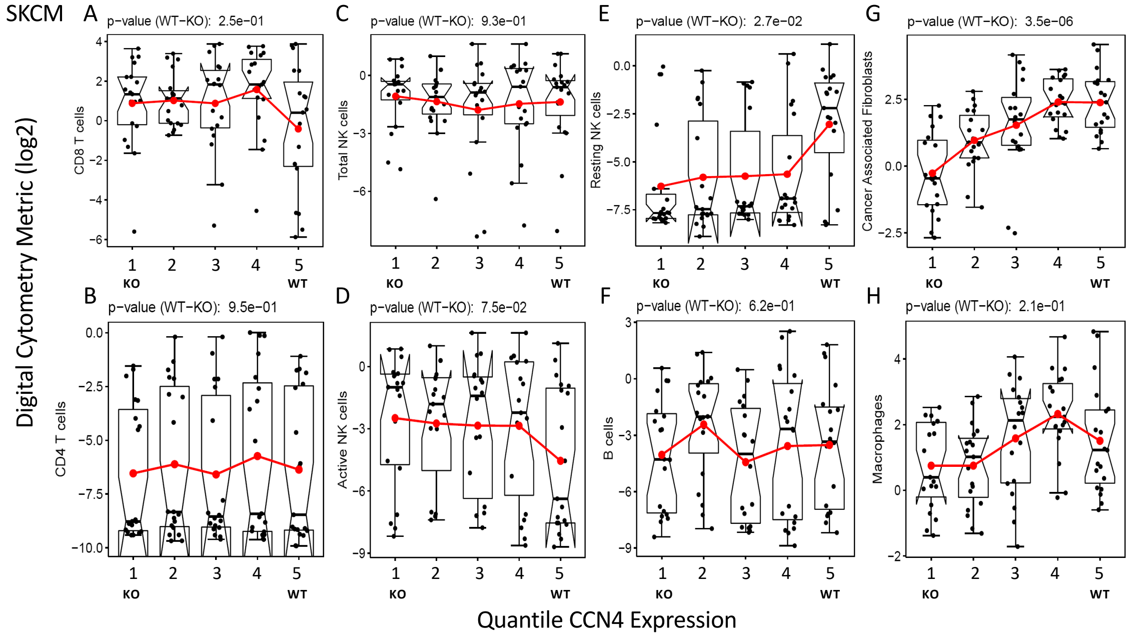

Supplementary Figure 9. Distribution in digital cytometry features extracted from the SKCM dataset stratified into quantiles based on CCN4 expression. The mean value for quantile 1 was used to represent CCN4 KO phenotype while quantile 5 was used to represent WT phenotype. A two-sided Wilcoxon test was used to assess significance. Summary quantile statistics from n = 94 biologically independent samples.are represented by the box-whisker plot, which include: upper whisker = the minimum of either the 75th percentile plus 1.5 times the inter-quartile range or the maximum value; upper box = 75th percentile; upper edge of notch = median + 1.57 times the inter-quartile range divided by the square root of n; cross bar = median; lower edge of notch = median - 1.57 times the inter-quartile range divided by the square root of n; lower box = 25th percentile; and lower whisker = the maximum of either the 25th percentile minus 1.5 times the inter-quartile range or the minimum value.

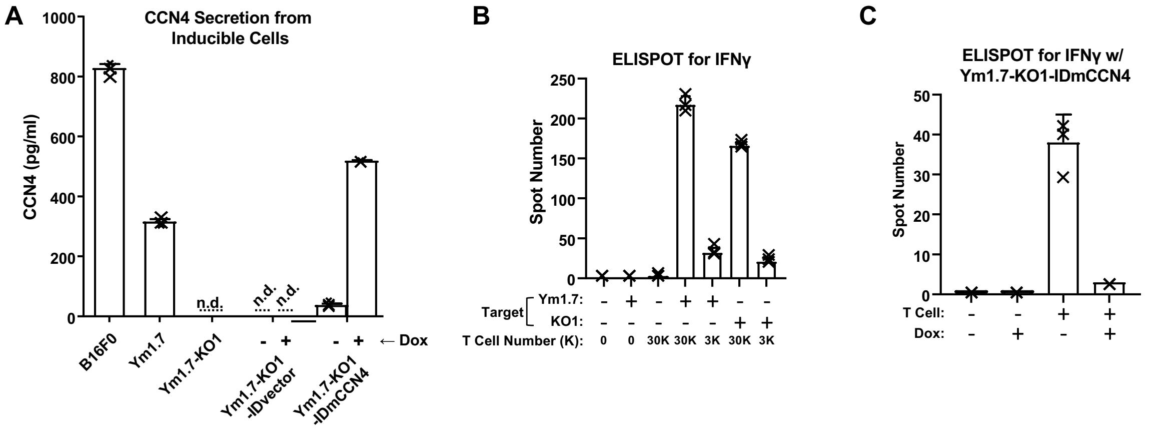

Supplementary Figure 10. Control experiments related to ELISpot assay using an inducible CCN4 YUMM1.7 cell line. (A) CCN4 secretion, measured with ELISA, from CCN4-inducible cells in conditioned media in the presence or absence of 0.5 μg/ml doxycycline (n.d. = not detected). (B) ELISpot for IFNγ release with different target cells and different amount of effector CD8$^+$ T cells (In vivo activated CD8$^+$ T cells against YUMM1.7 (Ym1.7)). (C) ELISpot for IFNγ with CCN4-inducible cells as targets using in vivo activated CD8+ T cell against YUMM1.7. Results shown as mean $\pm$ S.D. for three biological replicates.
