# Supplementary material for: Data-driven learning how oncogenic gene expression locally alters heterocellular networks
Source: Nat Commun. 2022 Apr 13;13:1986. doi: 10.1038/s41467-022-29636-3 (PMC9007999; doi:10.1038/s41467-022-29636-3)
Supplement: Supplementary file 2 — Reporting Summary [file 41467_2022_29636_MOESM2_ESM.pdf]

nature portfolio

Corresponding author(s): David J Klinke II

Last updated by author(s): Mar 17, 2022

Reporting Summary

Nature Portfolio wishes to improve the reproducibility of the work that we publish. This form provides structure for consistency and transparency in reporting. For further information on Nature Portfolio policies, see our Editorial Policies and the Editorial Policy Checklist.

Statistics

For all statistical analyses, confirm that the following items are present in the figure legend, table legend, main text, or Methods section.

n/a	Confirmed	
☐	☒	The exact sample size (*n*) for each experimental group/condition, given as a discrete number and unit of measurement
☐	☒	A statement on whether measurements were taken from distinct samples or whether the same sample was measured repeatedly
☐	☒	The statistical test(s) used AND whether they are one- or two-sided<br>*Only common tests should be described solely by name; describe more complex techniques in the Methods section.*
☒	☐	A description of all covariates tested
☐	☒	A description of any assumptions or corrections, such as tests of normality and adjustment for multiple comparisons
☐	☒	A full description of the statistical parameters including central tendency (e.g. means) or other basic estimates (e.g. regression coefficient) AND variation (e.g. standard deviation) or associated estimates of uncertainty (e.g. confidence intervals)
☐	☒	For null hypothesis testing, the test statistic (e.g. *F*, *t*, *r*) with confidence intervals, effect sizes, degrees of freedom and *P* value noted<br>*Give P values as exact values whenever suitable.*
☒	☐	For Bayesian analysis, information on the choice of priors and Markov chain Monte Carlo settings
☒	☐	For hierarchical and complex designs, identification of the appropriate level for tests and full reporting of outcomes
☐	☒	Estimates of effect sizes (e.g. Cohen's *d*, Pearson's *r*), indicating how they were calculated

Our web collection on statistics for biologists contains articles on many of the points above.

Software and code

Policy information about availability of computer code

Data collection	No custom code was used to collect data.
Data analysis	Microsoft Excel (version 16.57) was used to carry out simple data analysis operations. Flow cytometric data were acquired using FACSDiva software (version 8.0) and exported as FCS3.0 files and analyzed using R/Bioconductor (V3.5.1), as described in D. J. Klinke and K. M. Brundage. Scalable analysis of flow cytometry data using R/Bioconductor. Cytometry A, 75(8):699–706, Aug 2009.<br><br>Data analysis workflow for digital cytometry and Bayesian network inference was developed using R and can be obtained from the following GitHub repository: https://github.com/KlinkeLab/CellNetwork_2020 DOI: 10.5281/zenodo.6345206<br><br>Features corresponding to to the prevalence of endothelial cells, cancer-associated fibroblasts, macrophages, and CD4+ T cells were calculated using CIBERSORTx (https://cibersortx.stanford.edu) using the gene signatures derived from single cell RNAseq data while the prevalence of B cells naive, CD8+ T cells, Macrophage M0, Macrophage M1, Macrophage M2, activated NK cells, resting NK cells, and neutrophils were calculated using the LM22 immune cell gene signatures in CIBERSORTx run in absolute mode.

For manuscripts utilizing custom algorithms or software that are central to the research but not yet described in published literature, software must be made available to editors and reviewers. We strongly encourage code deposition in a community repository (e.g. GitHub). See the Nature Portfolio guidelines for submitting code & software for further information.

Data

Policy information about availability of data

All manuscripts must include a data availability statement. This statement should provide the following information, where applicable:

- Accession codes, unique identifiers, or web links for publicly available datasets
- A description of any restrictions on data availability
- For clinical datasets or third party data, please ensure that the statement adheres to our policy

The datasets supporting the conclusions of this article are available in Gene Expression Omnibus repository [https://www.ncbi.nlm.nih.gov] with the following GEO accession numbers: GSE98394.

Transcriptomics profiling of bulk tissue samples using Illumina RNA sequencing for the breast cancer (BRCA) and cutaneous melanoma (SKCM) arms of the Cancer Genome Atlas was downloaded from TCGA data commons, where values for gene expression were expressed in counts, using the "TCGAbiolinks" (V2.8.2) package in R (V3.6.1) and converted to TPM. This is detailed in the GitHub repository.

Field-specific reporting

Please select the one below that is the best fit for your research. If you are not sure, read the appropriate sections before making your selection.

☒ Life sciences ☐ Behavioural & social sciences ☐ Ecological, evolutionary & environmental sciences

For a reference copy of the document with all sections, see nature.com/documents/nr-reporting-summary-flat.pdf

Life sciences study design

All studies must disclose on these points even when the disclosure is negative.

Sample size	As this was an exploratory study, no sample-size calculation was performed prior to the study. However, independent experiments using multiple replicates are used to ensure reproducibility. The size of a cohort within a biological replicate was limited to the bandwidth of sample processing workflow.
Data exclusions	Mice were excluded from the study if they failed to develop tumors following subcutaneous challenge.
Replication	Experiments were repeated at least once, or data were compiled from two independent experiments. Replicates were reproducible.
Randomization	Mice were purchased from the indicated vendors, labeled and randomized to treatment groups/cages. Mice receiving different cell lines were housed in the same cages, at a density of five mice per cage.
Blinding	The investigators were not blinded to the group allocation during data collection or analysis, as the same individuals that set up the experiments were the ones that analyzed the results. They did remain objective in interpreting the data.

Reporting for specific materials, systems and methods

We require information from authors about some types of materials, experimental systems and methods used in many studies. Here, indicate whether each material, system or method listed is relevant to your study. If you are not sure if a list item applies to your research, read the appropriate section before selecting a response.

Materials & experimental systems

n/a	Involved in the study	
☐	☒	Antibodies
☐	☒	Eukaryotic cell lines
☒	☐	Palaeontology and archaeology
☐	☒	Animals and other organisms
☒	☐	Human research participants
☒	☐	Clinical data
☒	☐	Dual use research of concern

Methods

n/a	Involved in the study	
☒	☐	ChIP-seq
☐	☒	Flow cytometry
☒	☐	MRI-based neuroimaging

Antibodies

Antibodies used	In all assays, purified rat anti-mouse CD16/CD32 antibodies (Mouse BD Fc Block, BD Biosciences #553142) were used to block Fc receptors prior to antibody staining.<br><br>To analyze tumor-infiltrating T cells, the following anti-mouse antibodies and dyes were used: LIVE/DEAD Fix -- Violet/Pacific Blue

(Invitrogen #L34963); CD45 BB515 (clone: 30-F11, BD Biosciences #564590); CD3e Alexa Fluor 700 (clone: 500A2 BioLegend #152316); CD4 APC-Cy7 (Clone: GK1.5 BD Biosciences #552051); CD279 (PD-1) PE (Clone: REA802 BioLegend #135205); and CD8a APC (Clone: REA601 Miltenyi 130-109-248).

To analyze tumor-infiltrating NK and B cells, the following anti-mouse antibodies and dyes were used: LIVE/DEAD Fix -- Violet/Pacific Blue (Invitrogen #L34963); CD45 BB515 (clone: 30-F11, BD Biosciences #564590); CD3e Alexa Fluor 700 (clone: 500A2 BioLegend #152316); CD161 (NK-1.1) APC-Cy7 (Clone: PK136 BioLegend #108723); CD45R/B220 APC (Clone: RA3-6B2 BioLegend #103212); and CD49b PerCP/Cy5.5 (Clone: DX5 Biolegend #108915).

To analyze tumor-infiltrating myeloid cells, the following anti-mouse antibodies and dyes were used: LIVE/DEAD Fix -- Violet/Pacific Blue (Invitrogen #L34963); CD45 BB515 (clone: 30-F11, BD Biosciences #564590); CD11b PerCP/Cy5.5 (Clone: M1/70 eBioscience #45-0112-80); CD11c PE (Clone: N418 eBioscience #12-0114-81); F4/80 APC-Cy7 (Clone: BM8 BioLegend #123117); Ly-6G/Ly-6C (Gr-1) APC (Clone: RB6-8C5 BioLegend #108412); I-A/I-E (MHC-II) Alexa Fluor 700 (Clone: M5/114.15.2 BioLegend #107622).

To analyze the in vitro proliferation of T cells in the presence of tumor-conditioned medium, the following anti-mouse antibodies and dyes were used: CellTrace -- Pacific Blue ThermoFisher; LIVE/DEAD Fix -- Green/FITC (Invitrogen #L23101); CD4 APC-Cy7 (Clone: GK1.5 BD Biosciences #552051); CD8a APC (Clone: REA601 Miltenyi 130-109-248); CD62L PE (Clone: MEL-14 eBioscience #12-0621-82); and CD44 PerCP-Cy5.5 (Clone: IM7 BioLegend #103032).

Validation

All antibodies were purchased from the vendors mentioned above, where they are validated by the manufacturers and validation data are available at the manufacturers' websites. These antibodies are routinely used in our laboratory without additional validation.

Eukaryotic cell lines

Policy information about cell lines

Cell line source(s)

The mouse melanoma line B16F0 (purchased in 2008, RRID: CVCL_0604) and HEK293T (purchased in 2005, RRID: CVCL_0063) were obtained from American Tissue Culture Collection (ATCC, Manassas, VA). The mouse melanoma line YUMM1.7 (received in September 2017, RRID: CVCL_JK16) was a gift from Drs. William E. Damsky and Marcus W. Bosenberg (Yale University), who created the cell line. All cell lines were revived from frozen stock, used within 10-15 passages that did not exceed a period of 6 months.

Authentication

Cell lines were not authenticated.

Mycoplasma contamination

Cells were routinely tested for mycoplasma contamination by PCR, and found to be negative.

Commonly misidentified lines
(See ICLAC register)

None of the cell lines used were listed in the ICLAC database.

Animals and other organisms

Policy information about studies involving animals; ARRIVE guidelines recommended for reporting animal research

Laboratory animals

C57BL/6Ncrl mice (6-8 week-old, female) were purchased from Charles River Laboratories. Animals were housed with a 12-hour light/dark cycle (light 6 am to 6pm), temperature nominally 74 degrees F and humidity 50%. If temperature is out of the range of 68 to 79 or if the humidity is not in the range of 30 - 70%, it will alarm.

Wild animals

The study did not involve wild animals.

Field-collected samples

The study did not involve samples collected from the field.

Ethics oversight

All animal experiments were approved by West Virginia University (WVU) Institutional Animal Care and Use Committee and performed at the WVU Animal Facility (IACUC Protocol #1604002138).

Note that full information on the approval of the study protocol must also be provided in the manuscript.

Flow Cytometry

Plots

Confirm that:

- [x] The axis labels state the marker and fluorochrome used (e.g. CD4-FITC).
- [x] The axis scales are clearly visible. Include numbers along axes only for bottom left plot of group (a 'group' is an analysis of identical markers).
- [x] All plots are contour plots with outliers or pseudocolor plots.
- [x] A numerical value for number of cells or percentage (with statistics) is provided.

Methodology

Sample preparation

Subcutaneous tumors were surgically removed after euthanasia, weighted and processed into single cell suspensions using the Tumor Dissociation Kit, mouse (Miltenyi Biotec, Germany), according to the manufacturer's instructions. Briefly, the

tumors were cut into small 2-4 mm pieces, added to the enzyme mix, transferred to gentleMACS C-tubes (Miltenyi Biotec, Germany) and incubated in a gentleMACS Dissociator (Miltenyi Biotec, Germany) for the appropriated time at 37 DegC. In addition to following the manufacturer's instructions, the gentleMACS program 37C_m_TDK_1 was used for B16F0 tumors and 37C_m_TDK_2 was used for YUMM1.7 tumors. Following lysing of the red blood cells, the remaining single-cell suspensions were washed and stained with Live/Dead Fixable Pacific Blue Dead Cell Stain Kit (ThermoFisher). Following blocking with Mouse BD Fc Block (BD Biosciences), the surface of the cells were stained with one of three different antibody mixes that focused on T cells, NK and B cells, and myeloid cells and quantified by flow cytometry, where at least 500,000 events were acquired in each biological replicate.

Spleens were surgically removed after euthanasia, weighted and manually processed into single cell suspensions using sterile glass microscope slides. The resulting cell suspension was used to as a source of primary T cells for in vitro studies with flow cytometric readouts. In analyzing enriched cell populations, at least 20,000 events were acquired in each biological replicate.

Instrument

BD LSRFortessa, where the fluorescence intensity for each parameter was reported as a pulse area with 18-bit resolution and exported as FCS3.0 files.

Software

FACSDiva software (V8.0 BD Biosciences) was used to acquire flow cytometric data, where the fluorescence intensity for each parameter was reported as a pulse area with 18-bit resolution and exported as FCS3.0 files. Flow cytometric data analyzed using R/Bioconductor (V3.5.1), as described in D. J. Klinke and K. M. Brundage. Scalable analysis of flow cytometry data using R/Bioconductor. Cytometry A, 75(8):699–706, Aug 2009.

Cell population abundance

YUMM1.7-reactive CD8 T cells were isolated from splenocytes using a CD8a+ T cell isolation kit (Milteny Biotec #130-104-075) that uses magnetic beads. Enrichment of YUMM1.7-reactive cells was confirmed using functional ELISpot assays.

Gating strategy

The gating strategy is depicted in Supplementary Figs. S5 - S7 for T cells, NK/B cells, and myeloid cells, respectively.

☒ Tick this box to confirm that a figure exemplifying the gating strategy is provided in the Supplementary Information.
